# Surface Plasmon Resonance (SPR) Workflow for Comparative Analysis of Nanobody Variants Binding to Lysozyme as a Model Ligand

**DOI:** 10.1002/cpz1.70360

**Published:** 2026-04-29

**Authors:** Escarlet Díaz‐Galicia, Nicoleta Gutu, Yuli Peng, Almira Valitova, Dominik Renn, Magnus Rueping

**Affiliations:** ^1^ KAUST Catalysis Center (KCC), Division of Physical Sciences & Engineering King Abdullah University of Science and Technology, KAUST Thuwal Kingdom of Saudi Arabia; ^2^ Institute for Experimental Molecular Imaging, University Clinic RWTH Aachen University Aachen Germany

**Keywords:** nanobody, protein–protein interaction, single‐domain antibody, surface plasmon resonance (SPR), VHH

## Abstract

Developing protein interaction–based technologies such as biosensors requires a clear understanding of receptor‐target kinetics. Nanobodies, which are camelid‐derived single‐domain antibodies, are ideal biosensor receptors due to their high specificity, stability, and ease of production. During biosensor development, multiple nanobody variants are often tested against the same target to identify the best binders. Surface plasmon resonance (SPR) is a robust, label‐free method for measuring these interactions, but its many experimental variables can complicate the establishment of a streamlined protocol specially for non‐high‐throughput instruments. Here, we present an SPR workflow that enables the comparative analysis of nanobody variants binding to lysozyme as a model target on a Biacore T100 instrument. These protocols cover the steps from protein expression and purification to final affinity ranking. © 2026 The Author(s). *Current Protocols* published by Wiley Periodicals LLC.

**Basic Protocol 1**: Expression and purification of lysozyme‐specific nanobodies

**Support Protocol 1**: Protein identification

**Basic Protocol 2**: SPR workflow for the characterization of nanobody variants

**Support Protocol 2**: SPR planning and assay development

## INTRODUCTION

**Figure 1:**
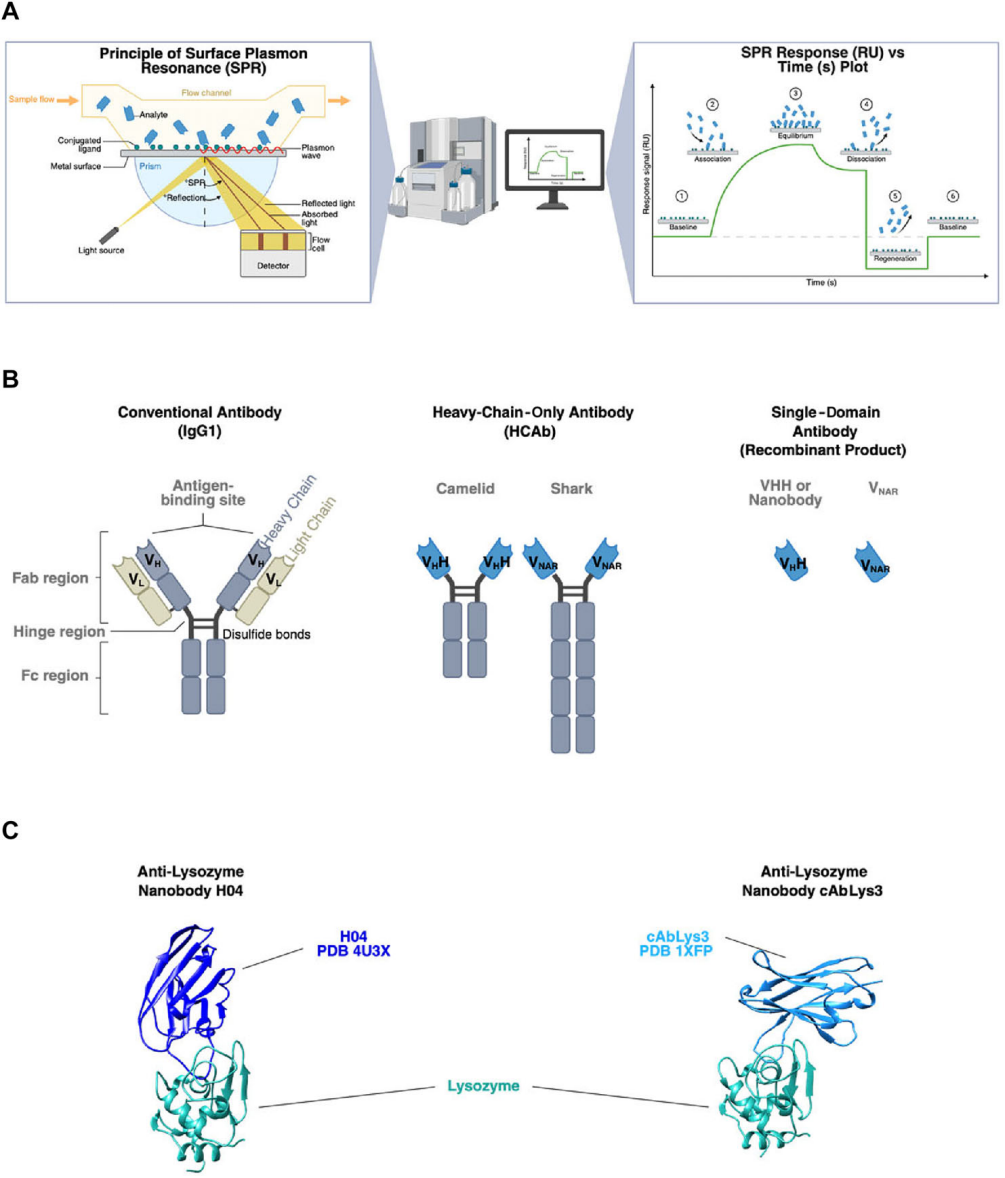
Surface plasmon resonance (SPR) working principle and nanobody origin and structure. (**A**) SPR instrument working principle and sensogram plot. (**B**) Depictions of the structure of conventional, heavy‐chain‐only, and single‐domain antibodies. (**C**) Structure of the model analytes: lysozyme‐binding nanobodies H04 PDB:4U3X and cAbLys3 PDB:1XFP. All images are created with BioRender.

Precise characterization of biomolecular interaction kinetics and affinities is essential for developing new therapeutics and affinity‐based diagnostics. Surface plasmon resonance (SPR) is a widely used optical technique that enables real‐time, label‐free monitoring of these interactions (Schasfoort, 2017) (Fig. 1A). SPR detects changes in the refractive index near a metal surface as molecules bind to an immobilized ligand, allowing determination of association and dissociation rate constants (*k*_on_, *k*_off_) as well as the equilibrium dissociation constant (*K*_D_) (Capelli et al., 2023; de Mol & Fischer, 2010). Nanobodies, single‐domain antibodies derived from the heavy chain–only antibodies of camelids, contain an antigen‐binding variable domain (VHH) that can be isolated to generate a small, stable, and fully functional antigen‐binding unit (Fig. 1B) (Hamers‐Casterman et al., 1993). Nanobodies are remarkably small (typically around 12 to 15 kDa), can be efficiently expressed in bacterial systems (De Meyer et al., 2014), can access hidden epitopes (De Genst et al., 2006), exhibit robust thermostability (Valdés‐Tresanco et al., 2023), have high tolerance to extreme pH and pressure conditions (De Vos et al., 2013; Jovčevska & Muyldermans, 2020), and retain strong antigen‐binding affinity, generally in the nanomolar to picomolar range (Pillay & Muyldermans, 2021). These properties make nanobodies suitable for applications in therapeutics (Blank et al., 2025; Duggan, 2018; Markham, 2022; Tanaka, 2023; Yu et al., 2024), imaging (Qi et al., 2024; Yu et al., 2024), and rapid diagnostics (Guo et al., 2021; Kang et al., 2025; Peng, Huang, et al., 2025; Peng, Alqatari, et al., 2025; Tanaka, 2023).

Reliable kinetic characterization is critical for evaluating nanobody binders. Multiple approaches exist for quantifying biomolecular interactions, each with its own advantages and limitations (Walport et al., 2021; Xu et al., 2025). However, SPR remains the most widely used technique for resolving association and dissociation kinetics in real time, particular for high‐affinity binders such as nanobodies (Capelli et al., 2023). As nanobody libraries expand, there is growing interest in high‐throughput kinetic analysis. However, adapting assays to higher throughput on instruments with inherently low‐throughput designs remains challenging (Feng et al., 2024; Yang et al., 2016). High‐quality SPR data depend on careful assay design, including sensor chip selection, immobilization strategy, buffer composition, ligand and analyte orientation, and regeneration conditions (Hojjat et al., 2025).

This article presents a practical SPR workflow for comparative kinetic analysis and ranking of nanobody binders against a single model antigen. It introduces core SPR concepts, including immobilization, referencing, and regeneration strategies, in a practical, hands‐on format and is designed so users can execute the workflow on any Biacore T100 SPR instrument. Step‐by‐step instructions span protein expression, purification, and assay setup.

Hen egg white lysozyme (HEWL) was selected as the model antigen due to its small size (14.5 kDa), stability, affordability, and widespread availability, making it ideal for assay development and benchmarking. Several nanobodies targeting HEWL have been reported across a range of affinities (Hassanzadeh‐Ghassabeh et al., 2013; Jovčevska & Muyldermans, 2020; Muyldermans, 2021; Revets et al., 2005; Vu et al., 1997). We selected the nanobodies cAbLys3 (Muyldermans & Lauwereys, 1999) and H04 (Rouet et al., 2015) to expand on their proposed use as standards in biophysical studies (Fig. 1C) (Birchenough et al., 2021). These well‐characterized nanobody‐HEWL interactions provide a robust reference system for validating the SPR workflow. Moreover, to facilitate the adoption of this set of protocols, plasmids encoding versions of these nanobodies with different purification tags (Strep‐Tag II or 6×His‐Tag) have been deposited in the Addgene repository [pET29b_HEWL‐VHH‐cAb‐Lys3‐6×His_1XFP (Addgene, no. 248353); pET29b_HEWL‐VHH‐H04‐6×His_4U3X (Addgene, no. 248574); pET29b_Strep‐HEWL‐VHH‐cAb‐Lys3‐Strep‐Tag II_1XFP (Addgene, no. 248575); and pET29b_Strep‐HEWL‐VHH‐H04‐Strep‐Tag II_4U3X (Addgene, no. 248576) (https://www.addgene.org/Magnus_Rueping/).

Basic Protocol 1 describes the expression and purification of lysozyme‐binding nanobodies. Support Protocol 1 details the steps to confirm the identity of the resulting proteins. Basic Protocol 2 provides the executable, easiest‐to‐reproduce SPR workflow for evaluating nanobody‐lysozyme binding, incorporating the assay development guidelines from Support Protocol 2.

## STRATEGIC PLANNING

**Figure 2:**
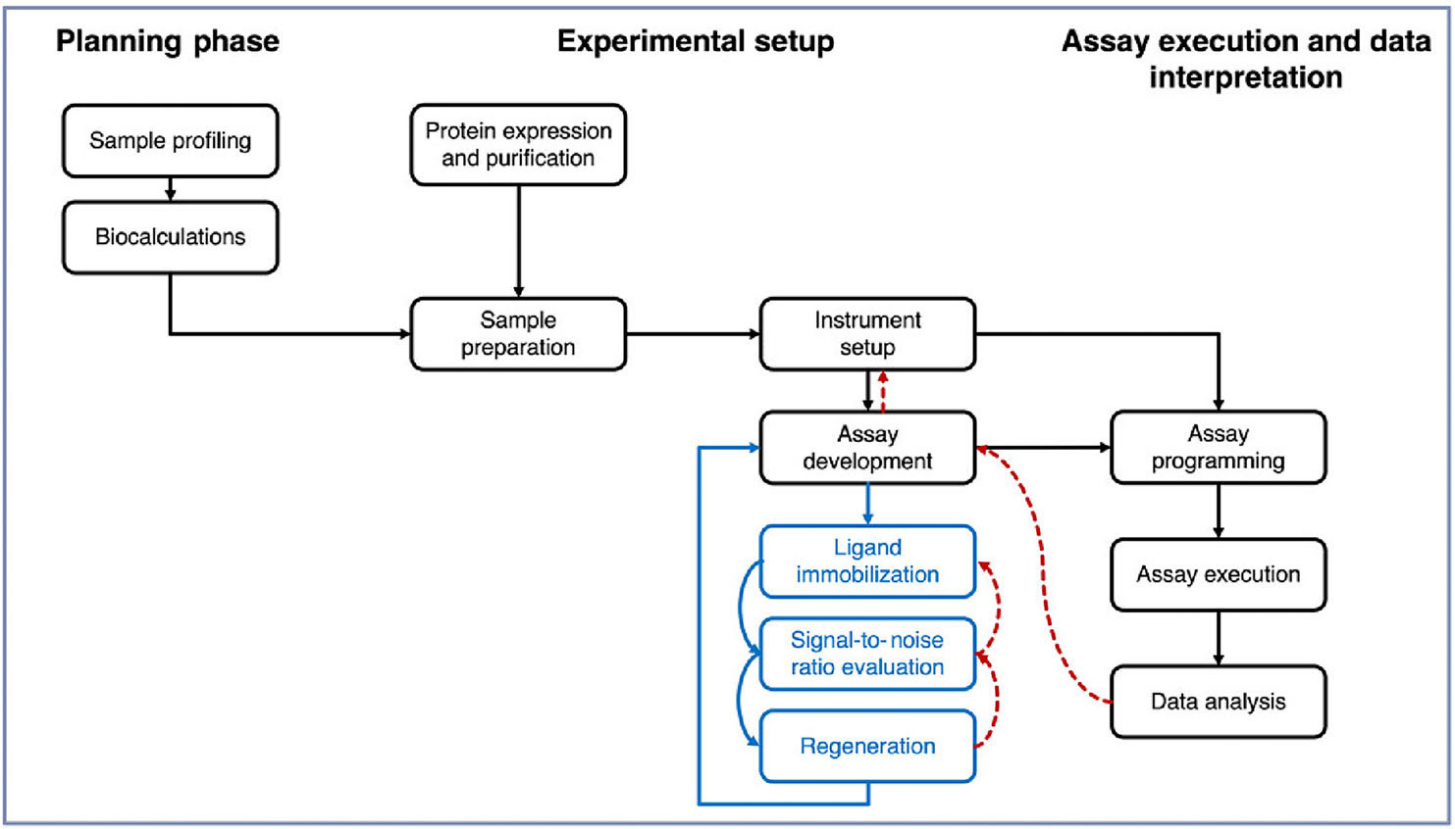
Flowchart illustrating the proposed surface plasmon resonance (SPR) workflow.

A successful SPR kinetic assay relies on four equally important stages: a) planning, b) assay development, c) assay execution, and d) data interpretation (Fig. 2). Even with careful planning, most kinetic assays require several iterations between these stages. The better the planning, the fewer iterations, errors, and costs and the more reliable the results. This workflow can be followed in two ways:

a) To directly reproduce the results for the reported characterization of lysozyme‐binding nanobody variants, follow Basic Protocols 1 and 2. Refer Support Protocol 2 only if you wish to gain a deeper understanding of the selected parameters.
b) To apply this workflow to new interactions, review the parameters and assay development described in the Support Protocols to adapt Basic Protocols 1 and 2 accordingly.

## Basic Protocol 1 EXPRESSION AND PURIFICATION OF LYSOZYME‐SPECIFIC NANOBODIES

Nanobodies are usually isolated from immunized or non‐immunized (i.e., naïve libraries) hosts or produced by *in silico* design (synthetic libraries) followed by experimental maturation or selection (Zhu et al., 2025). After identification, most nanobodies are produced recombinantly in prokaryotic, eukaryotic, and plant expression systems (Su et al., 2023). Due to its wide availability, rapid growth rate, low cost, and ease of transformation, *Escherichia coli* is one of the most preferred and utilized hosts to produce recombinant proteins (Rosano & Ceccarelli, 2014). Historically, nanobodies were expressed in and extracted from the bacterial periplasm; however, cytoplasmic expression can be 10 to 100 times more productive (Su et al., 2023). The *E. coli* SHuffle T7 strain carries mutations in the *trxB* and *gor* genes that promote an oxidizing cytoplasmic environment for the stable disulfide bond formation commonly found in nanobodies. Additionally, this cell line constitutively expresses a cytoplasmic version of the disulfide bond isomerase DsbC, which correctly rearranges non‐native disulfides. Together, these features promote a favorable environment for successfully producing disulfide‐bonded proteins, similar to many nanobodies, in the cytoplasm (Lobstein et al., 2012).

As adapted from Birchenough et al. (2021) with minor modifications, this protocol describes *E. coli* SHuffle T7 cytoplasmic expression and purification by affinity chromatography and size exclusion chromatography (SEC) of lysozyme‐binding nanobodies cAbLys3 (de Genst et al., 2004) and H04 (Rouet et al., 2015), both targeting the HEWL antigen. Both cAbLys3 nanobody (de Genst et al., 2004) and H04 nanobody (Rouet et al., 2015) have been engineered in two variants: one with a 6×His‐Tag and another with Strep‐Tag II at the C‐terminus of the protein sequence (see Supporting Information, Fig. S3; Addgene, no. 248353, 248574‐76).

### Materials

*E. coli* SHuffle T7 competent cells (New England Biolabs, cat. no. C3026J)
Expression plasmids:
  pET29b_HEWL‐VHH‐cAb‐Lys3‐6×His_1XFP (Addgene, no. 248353)
  pET29b_HEWL‐VHH‐H04‐6×His_4U3X (Addgene, no. 248574)
  pET29b_HEWL‐VHH‐cAb‐Lys3‐Strep‐Tag II_1XFP (Addgene, no. 248575)
  pET29b_HEWL‐VHH‐H04‐Strep‐Tag II_4U3X (Addgene, no. 248576)
SOC medium (see recipe), 30°C
LB agar plates with kanamycin (see recipe)
50 µg/ml kanamycin stock solution (see recipe)
LB culture medium (see recipe; make fresh)
1 M IPTG (see recipe)
Immobilized metal affinity chromatography (IMAC) binding buffer (see recipe)
Strep‐Tag II purification binding buffer (see recipe)

Lysis buffer (see recipe; make fresh)
2× sodium dodecyl sulfate (SDS) sample buffer (Invitrogen, cat. no. LC2676, or equivalent)
Distilled water
IMAC elution buffer (see recipe)
SEC buffer (see recipe)
Glycerol (Sigma‐Aldrich, cat. no. 49781)
Strep‐Tag II purification elution buffer (see recipe)
42°C water bath
28°C and 30°C temperature‐adjustable shaking incubators (Thermo Scientific, MAXQ6000, or equivalent)
30°C bacterial incubators
100‐ml and 2.5‐L baffled flasks
Cell density meter
Centrifuge bottles
Superspeed centrifuge (Thermo Scientific, Sorvall LYNX 6000 Superspeed Centrifuge, or equivalent), 4°C
5‐ml serological pipets
50‐ml Falcon tubes
Eppendorf centrifuge 5430 R or equivalent
Cell disruptor (Constant Systems CF1 or equivalent)
50‐ml Nalgene tubes
1.5‐ml Eppendorf tubes
Spectrophotometer (NanoDrop™)
ÄKTA chromatography system (Cytiva)
HisTrap column (HisTrap FF 5 ml, Cytiva, or equivalent)
Centrifugal filter units, 10‐kDa MWCO (Amicon Ultra Centrifugal Filter, 10 kDa, Millipore, cat. no. UFC9010)
Size exclusion column (Cytiva, HiLoad 16/60 Superdex 75 pg, or equivalent)
0.45‐µm syringe filters (optional)
96 deep‐well plate
StrepTrap HP column (StrepTrap HP 5 ml, Cytiva, or equivalent)

Additional reagents or equipment for SDS‐PAGE, western blot, and liquid chromatography–tandem mass spectrometry (LC‐MS/MS) (see Support Protocol 1)

#### Protein expression

1 Transform plasmids into *E. coli* SHuffle T7 competent cells:
  a. Thaw SHuffle T7 competent cell aliquots (50 µl) for each plasmid used for the transformation.
  b. Add 1 µl expression plasmid (containing 1 pg to 100 ng plasmid) to the competent cells and mix by gently pipetting.
  c. Incubate on ice for ≥20 min.
  d. Place the tube in a preheated 42°C water bath and heat shock the cells for exactly 30 sec.
  e. Immediately place the tube with cells on ice and incubate for 2 min.
  f. Add 450 µl pre‐warmed (30°C) SOC medium to cells and incubate at 30°C for 1 hr with shaking at 300 rpm in a shaking incubator.
  g. Warm LB agar plate with kanamycin at 30°C.
  h. Spread 50 and 150 µl cell suspension from each transformation onto two separate LB agar plates.
  i. Incubate the plates at 30°C overnight.
2 For the starter culture, add 10 µl of 50 µg/ml kanamycin stock solution to 10 ml LB culture medium in a 100 ml baffled flask. Pick one single colony from each transformed plate and grow in the 10 ml LB at 30°C overnight with shaking at 180 rpm.
3 Prepare production medium by adding 1 ml of 50 µg/ml kanamycin stock solution to 1 L LB culture medium in a 2.5‐L baffled flask, closing the flask, and shaking thoroughly.
4 Add the 10 ml starter culture from step 2 to the 1 L production medium from step 3.
5 Grow in 2.5‐L baffled flask at 30°C and 180 rpm in a shaking incubator until the OD_600_ reaches 0.6 to 0.7, with continuous measurement using a cell density meter.
6 Add 1 M IPTG to a final concentration of 0.8 mM.
  The same final concentration of IPTG is used for all plasmids mentioned in this protocol.
7 After induction, incubate the culture at 28°C for 24 hr with shaking at 180 rpm.
8 Pour the culture into a compatible centrifuge bottle and spin down in a superspeed centrifuge for 40 min at 6000 × *g*, 4°C.
9 Dispose of supernatant according to institutional waste disposal guidelines.
10 Resuspend the cell pellet in 30 to 40 ml IMAC binding buffer or Strep‐Tag II purification binding buffer (depending on the tagged variant) by pipetting up and down with a 5‐ml serological pipet and then pour the resuspension into a 50‐ml Falcon tube.
11 Centrifuge in Eppendorf centrifuge 5430 R or equivalent for 15 min at 6000 × *g*, 4°C.
12 Dispose of supernatant according to institutional waste disposal guidelines.
13 Freeze the pellet at −80°C or proceed with step 14.
14 If the pellet was frozen, thaw on ice and then resuspend the cells in 30 to 40 ml lysis buffer by pipetting up and down.
15 Lyse cells by a single passage through a cell disruptor at a pressure of 20 kPsi.
16 Centrifuge the lysate in the superspeed centrifuge for 1 hr at 20,000 × *g*, 4°C.
17 Carefully collect the supernatant in a 50‐ml Nalgene tube without touching the pellet and keep the cleared lysate on ice for purification.
18 In a 1.5 ml Eppendorf tube, collect 10 to 100 µl cleared lysate and dilute it with IMAC binding buffer or Strep‐Tag II purification binding buffer (depending on the tagged variant) to a final concentration of 20 mg/ml by measuring the absorbance at 280 nm on a spectrophotometer. Mix with 2× SDS sample buffer to obtain a final concentration of 10 mg/ml. Analyze this SDS sample by SDS‐PAGE (Support Protocol 1).

#### Ni‐NTA IMAC purification

19 Equilibrate ÄKTA chromatography system with IMAC binding buffer.
  Choose IMAC binding buffer for the nanobodies cAbLys3‐6×His and H04‐6×His.
20 Attach the HisTrap column to the ÄKTA. Wash the column with distilled water for 2 column volumes (CVs) and then equilibrate the column with IMAC binding buffer for 2 to 5 CVs.
21 Load supernatant of cAbLys3‐6×His or H04‐6×His nanobody from step 17 onto the column.
22 Wash the column with IMAC binding buffer for 5 CVs or until a stable UV baseline.
23 Apply 12 CVs of IMAC elution buffer over a gradient of 0% to 100%. Start fractionation and monitor the UV 280 nm signal.
  Choose IMAC elution buffer for the nanobodies cAbLys3‐6×His and H04‐6×His.
24 Collect all the fractions corresponding to the peak(s) in a 50‐ml Nalgene tube.
25 Estimate the concentration of the peak fractions by measuring with a spectrophotometer or integrating the peak area with the Äkta software.
26 In separate 1.5 ml Eppendorf tubes, prepare SDS samples by mixing an aliquot of each collected peak and 2× SDS sample buffer. Analyze the SDS samples by following Support Protocol 1. Estimate the final concentration, accounting for the dilution.
27 After analyzing the SDS‐PAGE gel, decide which peak fractions to select for SEC purification.
28 Concentrate the protein sample obtained after elution with a centrifugal filter unit of 10 kDa MWCO.
29 Equilibrate size exclusion column with SEC buffer at a flow rate of 1 ml/min for 1 to 1.5 CVs.
30 Before loading the protein into the injection loop, filter out possible aggregates by centrifugation (≥10 min at 14,000 rpm, 4°C) or by 0.45‐µm syringe filtration.
31 Purify the protein at a flow rate of 1 ml/min over 1.3 CVs in 1.8‐ml fractions in a 96 deep‐well plate.
32 Analyze peak fractions from SEC purification by SDS‐PAGE following Support Protocol 1.
33 Concentrate the fractions to the desired concentration and add glycerol to a final concentration of 20%. Store the protein sample at −20°C or −80°C.
34 Analyze purified proteins by SDS‐PAGE, western blot, and LC‐MS/MS following Support Protocol 1.

#### Strep‐Tagged protein purification

35 Equilibrate ÄKTA system with Strep‐Tag II purification binding buffer.
  Choose Strep‐Tag II purification binding buffer for the nanobodies cAbLys3‐Strep‐Tag II and H04‐Strep‐Tag II.
36 Attach the StrepTrap HP column to the ÄKTA. Wash the column with distilled water for 2 CVs and then equilibrate the column with Strep‐Tag II purification binding buffer for 2 to 5 CVs.
37 Load supernatant of cAbLys3‐Strep‐Tag II or H04‐Strep‐Tag II nanobody from step 17 onto the column.
38 Wash the column with Strep‐Tag II purification binding buffer for 5 CVs or until a stable UV baseline.
39 Apply 12 CVs of Strep‐Tag II purification elution buffer over a gradient of 0% to 100%. Start fractionation and monitor the UV 280 nm signal.
40 Follow steps 24 to 34.

## Support Protocol 1 PROTEIN IDENTIFICATION

Protein expression and purification are routinely monitored by SDS‐PAGE and western blot analysis, with protein sequence confirmation by LC‐MS/MS. This protocol describes the preparation of samples for SDS‐PAGE, followed by gel staining and imaging. It also provides a step‐by‐step description of immunoblot detection with anti‐6×His antibody and Strep‐Tactin. In addition, the protocol details downstream processing for LC‐MS/MS analysis, including excised gel band destaining, reduction, alkylation, tryptic digestion, and peptide desalting.

### Materials

Samples (see Basic Protocol 1)
2× SDS buffer (Novex Tris‐Glycine SDS Sample Buffer (2×), Thermo Fisher Scientific, cat. no. LC2676, or equivalent)
1× Tris‐glycine SDS buffer (Novex Tris‐Glycine SDS Running Buffer, Thermo Fisher Scientific, cat. no. LC26754, or equivalent)
Protein molecular‐weight marker (PageRuler Plus Prestained Protein Ladder, Thermo Fisher Scientific, cat. no. 26619, or equivalent)
Coomassie blue stain (Imperial Protein Stain, Thermo Fisher Scientific, cat. no. 24617; PageBlue Protein Staining Solution, Thermo Fisher Scientific, cat. no. 24620; or similar reagent)
Distilled water
Transfer buffer (Pierce 1‐Step Transfer Buffer, Thermo Fisher Scientific, cat. no. 84731 × 5, or equivalent)
Blocking buffer (Pierce Fast Blocking Buffer, Thermo Fisher Scientific, cat. no. 37575, or equivalent)
Antibody solution: anti‐6×His‐Tag antibody solution (see recipe; make fresh) or Strep‐Tactin HRP solution (see recipe; make fresh)
Wash buffer (Pierce Fast Wash Buffer, Thermo Fisher Scientific, cat. no. 37577, or equivalent)
Chemiluminescent reagents (Thermo Scientific, cat. no. 34580, or equivalent)
50 mM TEAB/50% (v/v) ACN (see recipe; make fresh)
100% acetonitrile (ACN; Sigma‐Aldrich, cat. no. 34851)
100 mM TEAB (see recipe)
1 M DTT (see recipe)
1 M IAA (see recipe)
10 ng/µl trypsin (see recipe; make fresh)
10% (v/v) TFA (see recipe)
1% (v/v) TFA/50% (v/v) ACN (see recipe; make fresh)
0.1% (v/v) TFA/75% (v/v) ACN (see recipe; make fresh)
0.1% (v/v) formic acid (see recipe)
95°C water bath or dry bath

Mini centrifuge
Precast or custom‐made 4%‐20% protein gels (Novex Tris‐Glycine Mini Protein Gels, 4%‐20%, 1.0 mm, WedgeWell format, Invitrogen, cat. no. 15466694)
Electrophoresis system, including gel box
Nitrocellulose transfer stacks (Power Blotter Pre‐cut Membranes and Filters, nitrocellulose, Invitrogen, cat. no. PB7320)
Black blot boxes or transparent trays
Orbital shaker
Semi‐dry transfer system (Invitrogen Power Blotter or equivalent)
Blotting roller
Aluminum foil
Imaging system
1.5 ml Eppendorf tubes
37°C thermomixer (Eppendorf ThermoMixer C)
37°C centrifugal vacuum concentrator
Vortex
MASCOT v2.3 software (Matrix Sciences)

Additional reagents or equipment for LC‐MS/MS (e.g., Orbitrap Fusion Lumos Tribrid Mass Spectrometer, Thermo Fisher Scientific)

#### SDS‐PAGE and gel staining

1 Prepare samples from Basic Protocol 1 by mixing with 2× SDS buffer, if not done already.
  If the same sample is to be analyzed by both gel staining and western blot, prepare enough sample to be loaded twice.
2 Denature samples for 5 min at 95°C in a water bath or dry bath if not done already.
3 Briefly spin down in a mini centrifuge to collect the condensate and cool to room temperature.
4 Insert a precast or custom‐made 4%‐20% protein gel into the electrophoresis system gel box.
5 Fill the upper and lower chambers with 1× Tris‐glycine SDS buffer.
6 Remove the comb from the gel and load samples (∼3 µg) together with the protein molecular‐weight marker.
7 Run the gel according to the manufacturer's instructions.
  After the run is complete, you might see faintly colored bands on the gel, confirming the success of labeling (if the dye absorbs highly in the visible range), but the best way to accurately detect is by using a fluorescence imager. Not all dyes will be visible on the unstained gel, as the visibility depends on the dye's properties and how well it binds to the protein of interest.
8 Proceed to stain the gel with Coomassie blue stain for appropriate time according to the manufacturer's instructions.
  If a portion of the gel is designated for immunoblotting, cut the gel into two sections: one for staining and the other for transfer onto a membrane.
9 Destain the gel with distilled water for appropriate time according to the manufacturer's instructions.

#### Western blot

10 Cut a nitrocellulose transfer stack (one nitrocellulose sheet and four filter papers) to the size of the gel portion or maintain the regular size of the stack (13 × 8.3 cm).
11 Pour transfer buffer into a black blot box or transparent tray, ensuring it will be enough to cover the nitrocellulose transfer stack and gel.
12 Submerge the gel portion designated for immunoblotting in the buffer.
13 Place transfer stack from step 10 on top of the gel in the transfer buffer.
14 Shake the box for 5 to 10 min at room temperature on an orbital shaker.
15 Unseal the cathode and anode stacks of the semi‐dry transfer system.
16 Place the nitrocellulose transfer stack in the upper middle of the anode stack (bottom cassette).
17 Remove two filter papers from the transfer stack and place the gel on top of nitrocellulose membrane. Ensure there are no bubbles between the membrane and gel by removing the bubbles using a blotting roller.
18 Place the two filter papers on top of the gel.
19 Use the blotting roller again.
20 Gently close the lid (cathode stack, top cassette) to ensure the transfer stack is not shifted from its position.
  If transferring more than one gel, ensure that there is a 1‐cm space around all stack edges.
21 Choose the appropriate method of transfer according to the size of protein of interest and the stack size following the manufacturer's instructions.
22 Pour blocking buffer in a black blot box or transparent tray. If using a transparent tray, cover it with aluminum foil to protect the fluorophore that is linked to the antibody (see step 27).
23 After transfer, briefly rinse the membrane with distilled water.
24 Submerge the membrane in blocking buffer.
25 Block the membrane in blocking buffer for 10 min with shaking.
26 Discard the blocking buffer and replace with HRP solution (anti‐6×His‐Tag antibody HRP solution or Strep‐Tactin HRP solution, depending on the tag of nanobodies).
27 Incubate the membrane in HRP solution for 1 hr at room temperature or overnight at 4°C.
  For detecting His‐Tagged nanobody, use the Anti‐6×‐His‐Tag antibody HRP solution; for detecting Strep‐Tagged nanobody, use the Strep‐Tactin HRP solution.
28 Remove the solution.
  At this point, the solution can be reused for approximately three more incubations.
29 Rinse the membrane two times with wash buffer for 10 min each with shaking on an orbital shaker.

#### Protein detection

30 Mix the chemiluminescent reagents at a 1:1 ratio and incubate with the membrane for 5 min.
  Around 2 ml each should be enough to cover the membrane, depending on how big the box or tray is.
31 Activate the imaging system and place the gel from step 9 over the white tray to image visible colorimetric protein.
  You have the option to image the gel in “Protein Fluorescence” mode. In this case, place the gel directly on the transilluminator glass.
32 Remove the gel.
33 Place the membrane from step 30 on the transilluminator glass.
34 Add a drop of water or wash buffer on top of membrane to keep it wet while imaging.
35 Assign dyes from the list according to the dye used for labeling and the dye attached to the anti‐6×His‐Tag antibody.
36 Image the membrane using the auto‐exposure setting and save the images.

#### Tryptic digestion

37 Cleave the band(s) of interest from the Coomassie blue–stained SDS‐PAGE gel from step 9.
38 Collect the gel pieces in a 1.5‐ml Eppendorf tube.
39 Completely cover the gel pieces with 50 mM TEAB/50% ACN.
40 Incubate with mixing in thermomixer at 37°C, 1000 rpm, for 3 min.
41 Discard the supernatant.
42 Repeat steps 39 to 41 several times, until the blue color is washed off.
  In the case that the color is hard to remove, overnight or longer washes are recommended.
43 Add 100% ACN and mix in thermomixer at 37°C, 1000 rpm, for 3 min.
  Note that at this step, all gel pieces should look white and be clumped together. Using 100% ACN dehydrates gel pieces, whereas 100 mM TEAB (see step 46) rehydrates gel pieces.
44 Discard the supernatant.
45 Dry the sample completely in a centrifugal vacuum concentrator at 37°C for 10 min.
46 Completely cover the gel pieces with 100 mM TEAB. Record the volume of added 100 mM TEAB.
  Always wait until the gel pieces rehydrate (indicated by swelling) and make sure all the gel pieces are covered.
47 Add 1 M DTT stock solution to a 10 mM DTT final concentration.
48 Vortex and quickly spin down.
49 Incubate at 37°C for 30 min in a prewarmed thermomixer.
50 Discard the supernatant.
51 Completely cover the gel pieces with 100 mM TEAB. Record the volume of added 100 mM TEAB.
52 Add 1 M IAA stock solution to 55 mM IAA final concentration.
  IAA solution must be kept in the dark at all times.
53 Vortex and quickly spin down.
54 Cover the tube with aluminum foil and incubate in the dark at room temperature for 1 hr.
55 Discard the supernatant.
56 Add 100 mM TEAB to completely cover the gel pieces.
57 Mix in thermomixer at 37°C, 1000 rpm, for 3 min.
58 Discard the supernatant.
59 Add 100% ACN to completely cover the gel pieces.
60 Mix in thermomixer at 37°C, 1000 rpm, for 3 min.
61 Discard the supernatant.
62 Repeat steps 56 to 61 once more.
63 Add 10 ng/µl trypsin to cover the dehydrated clumped gel pieces.
  Do not exceed 40 µl trypsin solution.
64 Wait until all gel pieces rehydrate and become clear again. If the trypsin cannot completely cover all the gel pieces, add 100 mM TEAB to just cover all the pieces.
  In this step, it is important not to add an excessive amount of trypsin because trypsin can repress peptide signals in MS analysis.
65 Incubate at 37°C overnight (12 to 16 hr) in a thermomixer.

#### Extraction of peptide from gel pieces and LC‐MS/MS

66 Add ∼20 µl of 10% TFA to inactivate trypsin.
  The final pH should be around 2 to 3.
67 Collect all supernatant in a new 1.5 ml Eppendorf tube.
68 Add 1% TFA/50% ACN to cover the gel pieces.
69 Vortex and quickly spin down.
70 Collect all supernatant in the tube from step 67.
71 Add 0.1% TFA/75% ACN to cover the gel pieces.
72 Vortex and quickly spin down.
73 Incubate at 37°C for 20 min in a thermomixer.
74 Collect all supernatant in the tube from step 67.
75 Repeat steps 68 to 74.
76 Dry the supernatant collected in the tube from step 67 in a centrifugal vacuum concentrator at 37°C until completely dried.
77 When ready to continue with LC‐MS/MS analysis, dissolve in 20 µl of 0.1% formic acid.
  Make sure no gel or dye particle is left in the sample. For deeper cleaning, use a ZipTip to clean and desalt the sample (for the procedure, see Current Protocols article: Stone & Williams, 2004). If the sample is not used immediately, store as dried powder at −20°C.
78 Submit the sample to a commercial MS facility or in‐house core lab or facility for LC‐MS/MS analysis.
79 Analyze data with MASCOT v2.3 software.

## Basic Protocol 2 SPR WORKFLOW FOR THE CHARACTERIZATION OF NANOBODY VARIANTS

This basic protocol describes the steps to set up an SPR kinetic analysis to compare variants of lysozyme‐binding nanobodies, namely, H04 and cAbLys3, from Basic Protocol 1. This workflow assumes that the planning and assay development have already been completed and then presents the minimum steps to reproduce the assay for the model interaction. Basic Protocol 2 can be further adapted to other molecular binding interactions by following the corresponding Support Protocol 2.

Lysozyme was immobilized on a CM5 chip via amine coupling, and the surface was then exposed to two tagged versions of each nanobody (tagged with 6×His or Strep‐Tag II). Multi‐cycle kinetic analysis, with regeneration between runs, enabled evaluation of the nanobodies’ binding profiles. The resulting sensogram and data analysis allowed clear differentiation between the tested anti‐lysozyme antibodies.

**Planning Summary**

This protocol describes the setup for comparing lysozyme‐binding nanobody variants (H04‐6×His, H04‐Strep‐Tag II, cAbLys3‐6×His, and cAbLys3‐Strep‐Tag II) against a common target, HEWL. Reported kinetic data from Birchenough et al. (2021) indicate dissociation constants (*K*_D_) of 19 ± 5 nM for H04‐6×His and 5 ± 4 nM for cAbLys3‐6×His. No values have been reported for the Strep‐Tagged variants. Based on this reference, the nanobody analytes are injected in a concentration series ranging from 3.3 to 1700 nM with a two‐fold dilution factor for H04 and from 0.6 to 300 nM with a two‐fold dilution factor for cAbLys3. Lysozyme (5 µg/ml in acetate buffer, pH 5.0) serves as the ligand to be immobilized on a CM5 sensor chip by amine coupling to a level of ∼150 RU. PBST is used as the running buffer, and all kinetic measurements are performed at room temperature. Regeneration cycles consist of one injection of 100 mM glycine, pH 2.2, for 5 s at a flow rate of 30 µl/min, followed by a 20‐s stabilization period before one injection of 10 mM NaOH for 5 s at 30 µl/min and then a 40‐s stabilization period. Resulting multi‐cycle kinetic sonograms will be analyzed for final nanobody comparison.

### Materials

BIAnormalization solution: 70% (w/w) glycerol
Desorb solution 1: 0.5% (w/v) SDS
Desorb solution 2: 50 mM glycine‐NaOH, pH 9.5
1× phosphate‐buffered saline (PBS; fresh)
Tween‐20
Lysozyme (from chicken egg white powder; Sigma‐Aldrich, 62971‐10G‐F)
Nanobody stock solutions (see Basic Protocol 1)
Distilled water, degassed and filtered (fresh)
50 mM NaOH
Immobilization buffer: 10 mM sodium acetate buffer, pH 5.5 (pH must be as accurate as possible)
100 mM glycine, pH 2.2
10 mM NaOH
70% (v/v) ethanol, degassed and filtered (optional)

Vacuum filtration unit (Nalgene reusable filter unit, Thermo Fisher, cat. no. 300‐4000)
Hydrophilic filter membrane, 0.45‐µm pore size, 47‐mm filter diameter (Millipore or equivalent)
1‐ to 2‐L glass beaker
Magnetic stirring bar
50‐ml Nalgene tubes
0.45‐µm syringe filters
Spectrophotometer (NanoDrop™)
1.5‐ml Eppendorf tubes
Refrigerated microcentrifuge (Eppendorf centrifuge 5430 R or equivalent), 4°C
Dialysis units, 10‐kDa MWCO (Slide‐A‐Lyzer)
Floater
Aluminum foil
Magnetic stirrer
Low‐binding Eppendorf tubes
Hydrophilic filter membrane, 0.45 µm, 47 mm (Milipore or equivalent)
Glass bottle
Biacore SPR T100 (Cytiva)
Maintenance chip
CM5 chip
BIAevaluation software

Additional reagents and equipment for preparing 0.4 M EDC, 0.1 M NHS, and 1 M ethanolamine‐HCl stock solutions (see recipes)

#### Experimental setup

1 Equilibrate the BIAnormalization solution and Desorb solutions 1 and 2 to room temperature.
  Be very careful to observe for precipitates. Filter if required.
  Temperature equilibration could take several hours.
2 Prepare 0.4 M EDC, 0.1 M NHS, and 1 M ethanolamine‐HCl stock solutions and freeze aliquots.

#### Sample preparation

3 Prepare fresh running buffer, consisting of 1 L fresh 1× PBS containing 0.01% Tween‐20 (PBST). Filter the solution using a vacuum filtration unit set with a 0.45‐µm pore‐size hydrophilic filter membrane. Transfer the filtered buffer into a 1 to 2‐L glass beaker and place a magnetic stirring bar at the bottom.
4 Prepare 100 µl of each sample (lysozyme and the nanobody variants) at a concentration of 25 µM:
  a. Lysozyme: In a 50‐ml Nalgene tube, dissolve 25 mg lysozyme in 5 ml running buffer. Filter through a 0.45‐µm syringe filter. Confirm the concentration by absorbance measurement using a spectrophotometer. Prepare 100‐µl aliquots in 1.5‐ml Eppendorf tubes. Freeze at −80°C.
  b. Nanobodies: Thaw the nanobody stock solutions on ice. Spin down for ≥15 min at 15,000 rpm, 4°C, to remove any aggregates. Immediately transfer the supernatant to a new tube and determine the protein concentration by absorbance measurement.
5 Load each protein sample into a dialysis unit (10‐kDa MWCO), avoiding contact with the membrane. Seal and label each unit properly. Place the dialysis units on a floater in 1 L of the prepared running buffer (see step 3). Cover the beaker with aluminum foil and start the magnetic stirrer at a medium speed to ensure gentle but consistent diffusion. Incubate at 4°C for 2 to 4 hr.
  Overnight incubation is possible if protein stability allows.
  Alternatively, desalting columns or ultrafiltration units can be used to equilibrate proteins in the running buffer, particularly when the stock concentration exceeds the highest assay concentration by more than 10‐fold. However, before optimization, the required protein amount is often uncertain. Additionally, desalting and ultrafiltration are more prone to incomplete buffer exchange, which can cause noisy sensograms. Hence, dialysis is preferentially selected to minimize baseline noise. Nonetheless, for proteins prone to precipitation during long dialysis times, these methods offer a practical alternative.
6 Recover dialysis samples. Once equilibration in the running buffer is complete, *do not discard this buffer* (see step 7). Remove the protein samples from the dialysis units and transfer them into clearly labeled low‐binding Eppendorf tubes. Spin down for ≥15 min at 15,000 rpm, 4°C, to remove any aggregates. Immediately transfer the supernatant to new tubes and determine the protein concentration by absorbance measurement. Keep the samples on ice until ready for instrument loading.
7 Recover dialysis buffer. Filter and degas the used dialysis buffer into a clean glass bottle with a vacuum filtration unit set with a hydrophilic filter membrane of 0.45‐µm pore size and 47‐mm filter diameter. Carefully aliquot 100 ml into two 50‐ml Nalgene tubes. Keep both at room temperature until use.

#### Instrument setup

8 Ensure that in both the left (line A) and right tray bottles of the Biacore SPR T100, there is 1 L fresh distilled water, filtered through a hydrophilic filter membrane of 0.45‐µm pore size and 47‐mm filter diameter and degassed with a vacuum filtration unit.
*IMPORTANT NOTE*: If the instrument is already prepared and switched on, proceed to step 23.
9 Close the pump clamp.
10 Switch on the PC.
11 Switch on the instrument from the rear panel.
12 Wait until the instrument status lights indicate it is ready.
  While waiting, listen for and note any unusual noises.
13 Launch the control software on the PC.
14 Ensure that the instrument is properly powered and connected.
  When the instrument is powered and connected, the label “COM” will appear in the lower‐left corner of the control software window.
15 Ensure that the instrument automatically opens the chip compartment door.
16 Insert a maintenance chip into the compartment, ensuring its correct orientation. Close the chip compartment door gently.
  Do not proceed to step 17 until the maintenance chip is inserted and the compartment door is closed.
17 In the software, select the chip type “Maintenance” and click “Dock.” Wait until the software recognizes the chip.
  The word “Maintenance” will appear in the lower‐left corner.
18 Set flow cell and sample compartment temperature to 25°C via Tools/Set Temperature.
19 Go to Tools/Prime. Prime three times with distilled water.
  Each prime cycle takes ∼7 min.
20 Select Tools/More Tools/Desorb and follow the on‐screen instructions.
  This method takes ∼16 min. Never perform Desorb on any chip other than the maintenance chip.
21 Open Manual Run.
22 Let the instrument to run for ≥1 hr.

#### Prime, mount chip, and check drift

23 Without taking the CM5 chip out of its packaging, allow it to equilibrate at room temperature for ≥30 min.
24 In the software, click “Stop standby,” wait until the flow stops, and then move line A from the distilled water bottle (left tray) into the running buffer (the same buffer used for sample dialysis).
25 Go to Tools/Prime. Prime three times with the running buffer.
  Each priming cycle takes ∼7 min.
26 Click “Eject chip” and wait until the instrument fully opens the chip compartment.
27 Remove the maintenance chip and insert the new CM5 chip that has been equilibrated to room temperature (see step 23). Mind the orientation. Close the chip compartment door gently.
  Do not proceed to step 28 until the chip is properly inserted and the compartment door is closed.
28 In the software, select the type of chip (CM5), click “Dock,” and wait until the instrument recognizes the chip.
  The label “CM5” will appear in the lower‐left corner of the window.
29 Go to Tools/Prime. Prime three times with running buffer.
  Each prime cycle takes ∼7 min.
30 Set the flow cell temperature to 25°C and the sample compartment temperature to 10°C via Tools/Set Temperature.
31 Navigate to Run/Manual Run. Activate two consecutive channels (either 1 and 2 or 3 and 4) and set a continuous flow of 15 to 30 µl/min for ≥2 hr (longer is preferable). Reference channel 2 against channel 1 or channel 4 against channel 3.
  An initial drift is expected but monitor the signal and note if significant drift (depends on the chip and instrument; <5 RU) persists beyond the first 60 min.
  From now on, this protocol will be simplified for the use of channels 1 (reference) and 2 (test), but it can be modified to test surface replicates by activating channels 3 and 4.

#### Ligand immobilization

32 Spin down the ligand stock (previously left on ice in step 6) for ≥15 min at 14,000 rpm, 4°C, to remove any possible aggregates. Immediately transfer the supernatant to a new tube and measure the protein concentration by absorbance measurement. Use the reserved running buffer (previously collected in 50‐ml Nalgene tubes in step 7) as the blank.
33 Stop the Manual Run in the instrument.
34 Open the Immobilization wizard: File > Open > NewWizardTemplate > Surface Preparation/Immobilization. Select CM5 chip. Activate flow cells 1 and 2. Select “Amine” coupling as the immobilization method for both channels.
  The reference channel (channel 1) must be activated as the test channel, but buffer will be used instead of ligand for its activation.
35 Choose “Blank” for the immobilization mode for channel 1.
36 Choose “Aim for immobilized level” for the immobilization mode for channel 2. Define a short name for the ligand (5 µg/ml lysozyme in 10 mM sodium acetate buffer, pH 5), for example, “5 µg/ml Lys in NaOAc pH 5”. Set target level to 150 RU. Set wash solution to 50 mM NaOH.
37 Make sure the unused channels are not activated.
38 Thaw 0.4 M EDC and 0.1 M NHS (see step 2) on ice immediately before use and keep them on ice (allocate ∼100 µl per channel).
39 Dilute the lysozyme stock to 5 µg/ml in immobilization buffer (10 mM sodium acetate buffer, pH 5).
40 Set up the vials in the sample rack according to the wizard's instructions: EDC, NHS, ligand (lysozyme diluted in immobilization buffer), ethanolamine‐Primary reference for the instrument Biacore T100HCl, BIAnormalization solution, and empty vials.
41 Double‐check that all samples are placed in the correct order and have the correct volume and confirm that the sample rack is properly positioned inside the compartment.
42 Run the immobilization wizard.
43 Activate the normalization option.
44 Verify that the ligand immobilization level matches the target response unit (RU) defined during assay development.

#### Assay execution

45 Open or create the assay program method (find .Method file).
46 Set the parameters in the method:
  a. In “General Settings,” make sure the sample compartment temperature is set to 8°C and the analysis temperature after run is 25°C.
  b. In “Assay Steps,” do the following:
    i. Make sure there are four main types of blocks: “Startup kinetics,” “Protein#_Kinetics,” “Binding control #” block repeated within each “Protein kinetics” block, and a “Clean up” cycle.
    ii. Ensure the block “Startup kinetics” is linked to the “Startup” purpose and connected to the “kinetics_control” cycle type. Select number of replicates.
    iii. Ensure each block “Protein#_Kinetics” is linked to the purpose “Sample” and connected to the respective “kinetics_sample#” cycle type.
    iv. For each “Binding control” block, ensure its purpose is “Control sample” and it is connected to the “kinetics_control” cycle type. Ensure that this block is within the “Protein#_Kinetics block” with a recurrence of “every 6 cycles.”
  c. In “Cycle types,” do the following:
    i. Ensure that the “kinetics_control” and the “kinetics_sample#” cycles are composed of the same three types of “capture”: “Sample #,” “Regeneration 1,” and “Regeneration 2.”
      There are three types of cycles: “kinetics_control”, “kinetics_sample #”, and “cleanup cycle”.
    ii. In “Sample #,” check “sample solution” as variable and define contact time of 180 s and dissociation time of 600 s for flow paths 1 and 2. Set flow rate to 70 µl/min.
    iii. Select “Regeneration 1.” Label the regeneration solution as “100 mM Glycine pH 2.2” Uncheck any method variables. Set contact time of 5 s for flow paths 1 and 2. Set flow rate to 30 µl/min.
    iv. Select “Regeneration 2.” Label the regeneration solution as “10 mM NaOH.” Uncheck any method variables. Set contact time of 5 s for flow paths 1 and 2. Set flow rate to 30 µl/min.
    v. For the “cleanup_cycle,” which contains only two “captures” (“Regeneration 1” and “Regeneration 2”), use the same parameters as indicated in step 46c, iii and iv.
  d. In “Variable settings,” enter the respective concentrations for each sample and define all values in the method:
    i. Startup kinetics: 0 nM PBST.
    ii. Protein1_kinetics: (H04‐6×His) two‐fold from 3.3 to 1700 nM, with two blanks at 0 nM concentration.
    iii. Protein2_kinetics: (cAbLys3‐6×His) two‐fold from 0.6 to 300 nM, with two blanks at 0 nM concentration.
    iv. Protein3_kinetics: (H04‐Strep‐Tag II) two‐fold from 3.3 to 1700 nM, with two blanks at 0 nM concentration.
    v. Protein4_kinetics: (cAbLys3‐Strep‐Tag II) two‐fold from 0.6 to 300 nM, with two blanks at 0 nM concentration.
    vi. Binding control: 0 nM PBST.
  e. In “Setup Run,” choose the detection flow path 2‐1,4‐3 and click “Next.” In the new window, select “Sample and reagent rack 2.” If required, adjust the location of the reagents.
47 Proceed to prepare the samples in the tubes and plate accordingly. Insert the rack into the instrument. Click “Next” Create an experiment name and define its location to save the results. Carefully read the program notes and verify that there is sufficient running buffer in the left tray, adequate distilled water in the right tray, and enough space in the waste bottle to accommodate the entire run.
48 Start the run.
  When modifications are made to the assay program, the software may automatically reassign reagent positions. Always verify the location of each reagent before starting the run to avoid errors or instrument damage.

#### Clean instrument for next user and leave running or switch off

49 Open the sample compartment and remove the sample tray.
50 Eject the assay chip and insert the maintenance chip back into the compartment.
51 Stop the flow before connecting a bottle with fresh, degassed distilled water in both left and right trays.
52 Prime the system twice with distilled water.
53 Perform a Desorb procedure on the instrument.
54 Stop the flow before removing the waste bottle. Empty it and then return it to its position as soon as possible.
55a *To leave instrument running*: If another user is scheduled to use the instrument or if advised by the facility manager, leave the instrument running with the maintenance chip installed, using either standby buffer or distilled water in line A.
55b *To switch off instrument*: If a full shutdown is required, follow the software switch‐off instructions to complete shutdown, which takes ∼20 min and requires ≥100 ml distilled water and 100 ml of 70% ethanol, both properly degassed and filtered.
  Always maintain at least a standby flow when the instrument contains buffer with salt or reagents prone to aggregation to prevent microfluidic cell blockage.

#### Data interpretation

56 Open the BIAevaluation software.
57 Import the sensogram from the run.
58 Select “Kinetics/Affinity” followed by “Surface bound”
59 Select the sample to be analyzed. Click “Next” to obtain the buffer‐subtracted curves.
60 Click “Kinetics” Select binding model “1:1 Binding” and click “Finish”
61 Wait for the instrument to iterate and produce the calculated fit.
62 Assess the quality of the fit by considering the quality parameters and report the results.
  SPR data validation involves checking the curves and fit but also several other key parameters. Residuals should be randomly distributed around zero, as systematic deviations indicate a poor fit. Chi‐squared (χ²) values are considered acceptable if <10% of R_max_. When available, the uniqueness value (U‐value) should be below 15 to ensure rate constants and R_max_ are not significantly correlated, though some instruments do not report this. For 1:1 binding models, the mass transport constant (t_c_) should be at least 100 times k_on_. Special caution is needed for very high‐affinity interactions with extremely low k_off_ (<1 × 10^−5^ s^−1^), as the dissociation may be slower than the instrument can reliably detect, making kinetic constants uncertain.
63 Repeat data interpretation steps for each sample.
64 Compare the results with the expected plots discussed in the “Understanding the results” section.

## Support Protocol 2 SPR PLANNING AND ASSAY DEVELOPMENT

A successful SPR kinetic assay (Basic Protocol 2) typically requires several rounds of optimization. Key parameters include the ligand immobilization strategy, the analyte concentration range, the regeneration conditions, and the association and dissociation times as well as the sample flow rate. This support protocol outlines the essential steps for planning and developing an SPR assay and uses a model interaction to exemplify the steps followed during assay development. Additional procedural details and background information can be found in the manufacturer's documentation and associated application guides, which served as references for this section (Biacore, 2005; Cytiva, 2020; GE Healthcare, 2001).

Biacore T100 offers three modes of operation that differ in flexibility and level of automation: Manual Run, Wizard Template, and Programmed Assay. For Manual Run, individual injections are directly controlled by the user. This method is useful for simple tasks like equilibration checks, regeneration scouting, testing immobilization conditions, or other early optimization steps. It provides limited automated scheduling but does not generate sensograms in a format readily analyzed by Biacore evaluation software (BIAevaluation). Wizard Templates are predefined workflows that guide the user through routine scouting or simple assays. These are less flexible for complex experimental designs. Data generated are compatible with BIAevaluation. Programmed Assay mode offers full customization and is suited for complex kinetic studies. However, its complexity requires careful programming to ensure that the assay is performed in the right order and that the resulting data integrate correctly with the evaluation software.

### Materials

See Basic Protocol 2.

#### Sample profiling and biocalculations

1 Define the purpose of the SPR protocol.
  In the example here, the purpose of this protocol is to compare among variants of lysozyme‐binding nanobodies. Specifically, it focuses on a common target, lysozyme, and four nanobody variants: H04‐6×His, H04‐Strep**‐**Tag II, cAbLys3‐6×His, and cAbLys3‐Strep**‐**Tag II. Other purposes, such as concentration analysis, epitope mapping, and other assays, require biocalculations beyond the purpose of this protocol.
2 Proceed to perform a literature search to define the expected *K*_D_.
  Birchenough et al. (2021) reported kinetic values for H04‐6×His (19 ± 5 nM) and cAbLys3 (5 ± 4 nM) in PBST buffer at room temperature. No available information was found for the Strep‐tagged versions of these nanobodies. In cases where there is no possible reference to define the expected K_D_, the analysis of a large range of concentrations is required. Most nanobodies have affinities in the nanomolar range, so one can start by assuming a K_D_ of about 1 to 10 nM and use this value to define the analyte serial dilution and, based on the preliminary results, iterate until finding the optimal range (best sensogram curve and fit).
3 Define running buffer and assay temperature.
  The running buffer is best selected based on the conditions under which one wants to understand the interaction under study but also needs to be compatible with the instrument and the method. Any reagent, even at low concentration, could precipitate and block the SPR flow cell or affect the bulk refractive index and produce artifacts. For the model interaction case, we aim to analyze the lysozyme‐nanobody interactions at room temperature and in 1× PBS, pH 7.4 + 0.05% Tween‐20 (PBST) as running buffer.
4 Assign ligand and analyte roles.
  There are two ways in which the model interaction experiment can be established: (a) defining the lysozyme as the ligand or the molecule to be immobilized over the chip or (b) passing over the lysozyme as the analyte. Although immobilizing either lysozyme or nanobody is theoretically expected to yield comparable binding parameters for the same interaction, in practice, the orientation, surface chemistry, and degree of ligand activity can influence the observed kinetics. For this reason, option (a) is simpler and more suitable for comparison purposes, as it allows different nanobody variants (targeting the same epitope) to be injected over the “same” lysozyme surface (assuming that, up to a limit, no substantial changes occur on the surface between analyte runs). However, one must monitor possible ligand detachment during long washing periods or surface deterioration after repeated regeneration cycles. Here, we observed no significant drift or reduction in ligand binding capacity after four analyte runs (data not shown). An additional advantage of option (a) is that only one chip is required, whereas the alternative orientation would typically require at least two.
5 Calculate optimal ligand immobilization level.
  The immobilization level directly influences the accuracy of the kinetic measurements and the determination of the binding parameters. An optimal ligand density can be estimated based on the molecular weights of the ligand and analyte, with the goal of achieving a response close to the theoretical maximum (R_max_) while minimizing mass transport limitations. The surface immobilization level (R_L_) can be calculated as follows:
$$\begin{array}{ccc} & & Surface\;immobilization\;level\;\left(R_{L}\right)=\\ & & \;\frac{Molecular\;weight\;of\;the\;ligand\;\left(MW_{L}\right)\times R_{max}}{Molecular\;weight\;of\;the\;analyte\;\left(MW_{A}\right)\times Stoichiometry\;} \end{array}$$
  The theoretical maximum response, or R_max_, is obtained when all immobilized ligand molecules are occupied by the analyte. It is determined by the ligand and analyte molecular weights, surface immobilization level or ligand density (R_L_), and binding stoichiometry and is independent of instrument sensitivity. For protein‐protein interactions, an R_max_ in the range of 50 to 150 RU generally provides sufficient signal without introducing mass transport effects. In our model system, the four nanobody variants have similar molecular weights (∼15 kDa), which allowed us to target an R_L_ of ∼150 RU, which yields an R_max_ of ∼150 RU (Table 1). This target, however, may need adjustment during assay development for other binding interactions. When analytes differ substantially in molecular weight, a more detailed evaluation is required to select a ligand density high enough to maintain sensitivity (i.e., for low‐molecular‐weight analytes) yet low enough to avoid mass transport limitations, which occur when the analyte diffusion to the sensor surface becomes slower than its binding rate or rebinding artifacts. For interactions with stoichiometries other than 1:1, more iterations may be required to identify the optimal ligand immobilization level.

**Table 1:** Amount of Immobilized Ligand by Interaction Pair

Ligand	Ligand molecular weight (MW_L_) (g/mol)	Analyte	Analyte molecular weight (MW_A_) (g/mol)	Stoichiometry	*R*_max_ (RU)	Immobilized ligand (*R*_L_) (RU)
Lysozyme	14,600	H04‐6×His	14,200	1:1	150	154.2
Lysozyme	14,600	cAbLys3‐6×His	15,200	1:1	150	144.1
Lysozyme	14,600	H04‐Strep‐Tag II	14,878	1:1	150	147.2
Lysozyme	14,600	cAbLys3‐Strep‐Tag II	15,638	1:1	150	140.0

#### Experimental setup

6 Verify protein availability.
  Set aside additional protein for assay development, including scouting steps (immobilization tests, regeneration scouting, concentration range adjustments), as new assays typically require exploratory injections prior to final method setup. We recommend ensuring that ≥100 µl of each protein at ≥100 µM is available before starting the assay. However, much less sample is usually required for an already developed assay.
7 Confirm storage buffer composition.
  Review the buffer in which each protein is stored and verify its compatibility with both the running buffer and the immobilization chemistry.
8 Plan sample preparation time.
  Allocate sufficient time for buffer exchange, concentration adjustment, or filtration as needed (usually 1 day is enough). Also reserve sufficient time for instrument usage (several days for assay development, usually 1 or 2 days for already developed assays).

#### Assay development

9 Determine suitable immobilization, generation, and binding parameters.
  The SPR instrument is user friendly: you load the reagents into the correct vials and racks, and it performs precise, automated injections at set times and flow rates. However, method setup still requires exploratory work. SPR experiments require careful optimization at each step, and this is where user expertise matters most. Whenever you use new analytes, ligands, chips, buffers, or assay conditions, you must first scout suitable immobilization, regeneration, and binding parameters before programming a reliable assay.

#### Ligand immobilization

10 Choose the assay sensor chip.
  For the Biacore T100, the sensor chip is composed of a cassette that protects a polystyrene support sheath where a gold‐coated glass slide is mounted. There are several types of sensor surfaces (Table 2) (Cytiva, https://www.cytivalifesciences.com/en/us/products/category/protein‐analysis/spr‐label‐free‐analysis/spr‐consumables/spr‐sensor‐chips). The gold standard is the CM5 chip, which is composed of a carboxylmethylated dextran matrix. For the purposes of Basic Protocol 2, we selected the CM5 chip.

**Table 2:** Summary of Chip Sensors Available for Biacore T100

Chip	Description	Usage	Ligand coupling strategy	Regeneration
Au	Bare gold surface	Development of custom surface chemistries	Depends on user‐defined chemistry	Depends on user‐defined chemistry
C1	Flat carboxymethylated surface without dextran (∼10% capacity of CM5)	For assays avoiding dextran; large analytes (cells, viruses); comparison to CM5	Standard amine coupling principles as CM5	Same as CM5; conditions depend on ligand stability
CM3	Short dextran matrix, similar charge to CM5	Large analytes; exploratory assay formats	Covalent coupling to carboxyl groups on dextran (amine, thiol, aldehyde, hydroxyl, carboxyl chemistries)	Selective analyte dissociation without affecting ligand
CM4	Lower carboxylation; dextran like CM5 but lower charge	Reduces nonspecific binding; suitable for low ligand densities	Covalent coupling via dextran carboxyl groups	Selective analyte dissociation
CM5	Standard carboxymethylated dextran matrix	General‐purpose chip for most biomolecules	Covalent coupling via dextran carboxyl groups	Selective analyte dissociation
CM7	High carboxylation; ∼3× capacity of CM5	High‐capacity capture; suitable for fragments and small molecules	Covalent coupling via dextran carboxyl groups	Selective analyte dissociation
HPA	Long‐chain alkane thiol monolayer (hydrophobic surface)	Liposome adsorption and formation of supported lipid monolayers	Hydrophobic adsorption of liposomes	Remove analyte while retaining lipid monolayer; lipids tolerate up to 100 mM NaOH/HCl (ligand‐dependent)
L1	CM dextran with lipophilic groups	Rapid capture of lipid vesicles ± membrane proteins	Vesicle adsorption	Vesicle stripping or selective analyte dissociation
NTA	CM dextran with nitrilotriacetic acid (NTA)	Capture of His‐Tagged ligands	Ni²⁺–NTA chelation of His‐tag	Removal of Ni²⁺ and chelated molecules; recharge with Ni²⁺
PEG	Flat PEG‐modified SAM surface	Alternative to dextran; reduces nonspecific binding; suited for large/multivalent analytes	Amine coupling	Selective analyte dissociation
Protein A	CM dextran with recombinant Protein A	Oriented capture of IgG via Fc region	Noncovalent reversible Fc capture	Selective analyte dissociation (ligand remains bound)
Protein G	CM dextran with recombinant Protein G	Capture of antibodies from various species/subclasses	Noncovalent reversible Fc capture	Removal of captured ligand + analyte (surface regeneration)
Protein L	CM dextran with recombinant Protein L	Capture of antibodies/fragment with κ‐light chains (e.g., Fab, scFv, Dabs)	Noncovalent reversible κ‐light chain capture	Removal of captured ligand + analyte
SA (Streptavidin)	CM dextran with streptavidin	High‐affinity capture of biotinylated ligands	Irreversible biotin‐streptavidin capture	Remove analyte (ligand remains bound)
CAP	Surface with immobilized DNA oligos	Capture of biotinylated ligands via CAP reagent	Hybridization of CAP reagent, followed by biotinylated ligand capture	Disrupt oligo hybridization
NA (NeutrAvidin)	CM dextran with NeutrAvidin	Alternative to covalent coupling; oriented capture via controlled biotinylation	High‐affinity biotin capture	Remove analyte (ligand remains bound)

11 Define the analyte concentration curve.
  Determine the analyte concentration range based on the expected K_D_, typically spanning 10× to 20× above and below this value. Generally, a two‐fold (1:2) serial dilution is suggested. The exact range and number of concentrations may be adjusted depending on assay requirements, number of replicates, and available analyte stock.
12 Prepare the sensor chip surface:
  a. Select a type of coupling.
    There are several ways to immobilize a ligand over the SPR surface. This depends (a) on the chip surface and (b) on the coupling method.
    Ligand immobilization strategies depend on the nature of the interaction between the ligand and the sensor surface and can be broadly classified into three categories: direct adsorption onto hydrophobic or bare gold surfaces, covalent immobilization, and noncovalent affinity capture. Covalent immobilization includes chemistries such as amine coupling via EDC/NHS activation of surface carboxyl groups, as well as thiol‐, aldehyde‐, hydroxyl‐, and carboxyl‐directed coupling, whereas noncovalent capture relies on affinity interactions such as Ni²⁺‐NTA, biotin‐streptavidin, or Fc/κ–light chain binding. Alternative approaches include pre‐immobilized surfaces and hybrid methods, such as CAP chips (Capelli et al., 2023; GE Healthcare, 2001; see Current Protocols article: Jason‐Moller et al., 2006).
  b. Scout to select the best immobilization buffer.
    i. Prepare ligand solutions in different coupling buffers to be tested.
      The suitable buffer (pH and composition), temperature, flow rate, and concentration for ligand immobilization will be determined.
    ii. Run the Wizard Template “Immobilization scout”
      During “Immobilization scout,” the ligand is injected in coupling buffer over a non‐activated sensor surface to assess suitable coupling conditions without permanently modifying the chip. Transient noncovalent electrostatic interactions (pre‐concentration) are observed as an increase in response and are used to evaluate whether the coupling conditions are appropriate. As a general guideline, effective pre‐concentration typically results in a ligand response of ≥5000 RU above baseline within 2 min of injection (Capelli et al., 2023; GE Healthcare, 2001; see Current Protocols article: Jason‐Moller et al., 2006). Pre‐concentration scouting cannot be performed on all sensor chip types. Depending on the chip chemistry and immobilization strategy, each iteration may require the use of a new sensor chip.
    iii. Choose the highest pH that gives adequate pre‐concentration.
      In the case of the model interaction, a 10 mM sodium acetate buffer with pH 5.0 was the best.
13 Scout regeneration conditions:
  a. Inject analyte over the immobilized surface.
    The conditions for regeneration are dictated by the nature of the ligand‐analyte interaction and the stability of the ligand and analyte and the capturing approach used. Different applications need individual tailored regeneration conditions. The regeneration scout can be performed with Manual Run or Wizard Template modes.
  b. Monitor the binding response to confirm successful association.
  c. Inject the chosen regeneration solution and injection plan (single or sequential injections of the same or different regeneration solutions). Begin with mild regeneration conditions. Select the least harsh buffer or solution expected to remove the analyte without affecting the ligand.
  d. Observe the response decrease after injection.
  e. Evaluate analyte removal.
    If the post‐regeneration baseline returns to the pre‐injection level, regeneration is likely sufficient. If the analyte remains, proceed to a stronger regeneration condition.
  f. Test ligand activity. Inject analyte again after regeneration.
  g. Compare the binding response to the initial analyte injection.
    If activity is reduced, the regeneration condition may be too harsh.
  h. Iteratively increase regeneration strength.
  i. Progressively adjust pH, ionic strength, contact time, or additive concentration. Choose the mildest condition that fully removes analyte while maintaining ligand activity across cycles.
    Typical regeneration conditions/solutions are as follows:
    - Brief exposure to acidic (glycine‐HCl buffer or diluted HCl) or basic (NaOH) solution
    - Solution with high ionic strength (i.e., 1 to 2 M NaCl or 1 to 4 M MgCl_2_)
    - Up to 100% ethylene glycol
    - Low concentrations of SDS (e.g., 0.05%)
    - 20 to 100 mM NaOH in 30% ACN (particularly useful for regeneration of low‐molecular‐weight ligands)
      Care must be taken to confirm the regeneration solution is compatible with the running buffer to avoid precipitation issues.

## REAGENTS AND SOLUTIONS

### 0.1% TFA/75% ACN

0.1 ml 10% (v/v) TFA (see recipe)
7.5 ml 100% ACN (Sigma‐Aldrich, cat. no. 34851)
2.4 ml HPLC‐grade H_2_O
Prepare fresh immediately before use

### 1% TFA/50% ACN

1 ml 10% (v/v) TFA (see recipe)
5 ml 100% ACN (Sigma‐Aldrich, cat. no. 34851)
4 ml HPLC‐grade H_2_O
Prepare fresh immediately before use

### 50 mM TEAB/50% ACN

5 ml 100 mM TEAB (see recipe)
5 ml 100% ACN (Sigma‐Aldrich, cat. no. 34851)
Prepare fresh immediately before use

### Anti‐6×His‐Tag antibody solution

5 ml Pierce Fast Blocking Buffer (Thermo Fisher Scientific, cat. no. 37575)
2 µl 1 mg/ml 6×‐His‐Tag Monoclonal Antibody (HIS.H8), HRP (Thermo Fisher Scientific, cat. no. MA1‐21315‐HRP, or equivalent)
Prepare fresh immediately before use and store at 4°C for three more incubations

### DTT, 1 M

0.15 g dl‐dithiothreitol (DTT; Sigma‐Aldrich, cat. no. 43817)
1 ml 100 mM TEAB (see recipe)
Store ≤6 months at −20°C

### EDC, 0.4 M

Dissolve 750 mg EDC [1‐ethyl‐3‐(3‐dimethylaminopropyl) carbodiimide hydrochloride] into 10 ml distilled H_2_O
Vortex until solids are dissolved completely
Aliquot and store ≤2 months at −20°C

### Ethanolamine‐HCl, 1 M

Dissolve 610.8 mg ethanolamine hydrochloride (MW 61.08 g/mol; Sigma, cat. no. E6133, CAS 2002‐24‐6) in 8 ml distilled H_2_O
Adjust pH to 8.5 by adding NaOH
Mix well
Distilled H_2_O to 10 ml final volume
Filter using 0.45‐µm filter
Aliquot and store ≤6 months at −20°C
Formic acid, 0.1%

0.01 ml 100% formic acid (Sigma‐Aldrich, cat. no. 5.33002)
9.99 ml HPLC‐grade H_2_O
Store ≤2 weeks at room temperature

### IAA, 1 M

0.18 g iodoacetamide (IAA; Sigma‐Aldrich, cat. no. I6125)
1 ml 100 mM TEAB (see recipe)
Store ≤1 month at −20°C in the dark, covered with aluminum foil

### IMAC binding buffer

8 g sodium chloride (136.893 mM; Sigma‐Aldrich, cat. no. S3014, or equivalent)
0.2 g potassium chloride (2.683 mM; Sigma‐Aldrich, cat. no. 529552, or equivalent)
1.44 g sodium phosphate dibasic (10.144 mM; Millipore, cat. no. 567547, or equivalent)
0.245 g potassium phosphate monobasic (1.8 mM; Millipore, cat. no. 529568, or equivalent)
1.36 g imidazole (20 mM; Sigma‐Aldrich, cat. no. I2399, or equivalent)
Distilled H_2_O to 0.8 L
Adjust to pH 7.0 by adding either HCl or NaOH
Mix well
Distilled H_2_O to 1 L
Filter using 0.45‐µm filter
Degas using vacuum filtration unit
Store ≤1 month at 4°C

### IMAC elution buffer

8 g sodium chloride (136.893 mM; Sigma‐Aldrich, cat. no. S3014, or equivalent)
0.2 g potassium chloride (2.683 mM; Sigma‐Aldrich, cat. no. 529552, or equivalent)
1.44 g sodium phosphate dibasic (10.144 mM; Millipore, cat. no. 567547, or equivalent)
0.245 g potassium phosphate monobasic (1.8 mM; Millipore, cat. no. 529568, or equivalent)
34.04 g imidazole (500 mM; Sigma‐Aldrich, cat. no. I2399, or equivalent)
Distilled H_2_O to 0.8 L
Adjust to pH 7.0 by adding either HCl or NaOH
Mix well
Distilled H_2_O to 1 L
Filter using 0.45‐µm filter
Degas using vacuum filtration unit
Store ≤1 month at 4°C

### IPTG, 1 M

2.38 g IPTG (isopropyl β‐d‐1‐thiogalactopyranoside; PanReac AppliChem, cat. no. 1008, or equivalent)
Distilled H_2_O to 1 L
Filter‐sterilize using 0.22‐µm syringe filter
Make 1 ml aliquots
Store ≤1 month at −20°C

### Kanamycin stock solution, 50 µg/ml

0.5 g kanamycin sulfate (Fisher Bioreagents, cat. no. 25389‐94‐0, or equivalent)
Distilled H_2_O to 10 ml
Filter‐sterilize using 0.22‐µm syringe filter
Make 1‐ml aliquots
Store ≤1 year at −20°C

### LB agar plates with kanamycin

32 g LB agar powder (Invitrogen, cat. no. 22700041)
Distilled H_2_O to 1 L
Autoclave
Wait until temperature decreases to 55°C
Add 1 ml 50 µg/ml kanamycin stock solution (see recipe) and mix well
Pour into sterile 90‐mm petri dishes
Wait until LB agar solidifies
Store ≤3 months at 4°C

### LB culture medium

20 g LB base powder (Invitrogen, cat. no. 12780029, or equivalent)
Distilled H_2_O to 1 L
Autoclave
Prepare fresh immediately before use

### Lysis buffer

50 ml IMAC or Strep‐Tag II purification binding buffer (see recipes)
50 µg deoxyribonuclease I (10 µg/ml; Sigma‐Aldrich, cat. no. DN25)
1 protease inhibitor tablet (Thermo Fisher Scientific, cat. no. A32965)
50 µl 1 M MgCl_2_ solution (Sigma‐Aldrich, cat. no. M1028, or equivalent)
Prepare fresh immediately before use and keep at 4°C

### NHS, 0.1 M

Dissolve 115 mg NHS (N‐hydroxysuccinimide) into 10 ml distilled H_2_O
Vortex until solids are dissolved completely
Aliquot and store ≤2 months at −20°C

### SEC buffer

8 g sodium chloride (136.893 mM; Sigma‐Aldrich, cat. no. S3014, or equivalent)
0.2 g potassium chloride (2.683 mM; Sigma‐Aldrich, cat. no. 529552, or equivalent)
1.44 g sodium phosphate dibasic (10.144 mM; Millipore, cat. no. 567547, or equivalent)
0.245 g potassium phosphate monobasic (1.8 mM; Millipore, cat. no. 529568, or equivalent)
Distilled H_2_O to 0.8 L
Adjust to pH 7.4 by adding either HCl or NaOH
Mix well
Distilled H_2_O to 1 L
Filter using 0.45‐µm filter
Degas using vacuum filtration unit
Store ≤1 month at 4°C

### SOC medium

2 g peptone [2% (w/w); Fluka Analytical, cat. no. 87972, or equivalent]
0.5 g yeast extract [0.5% (w/w); Fluka Analytical, cat. no. 92144, or equivalent]
0.06 g NaCl (10 mM; VWR Chemicals, cat. no. 27810.460, or equivalent)
0.02 g KCl (2.5 mM; Sigma‐Aldrich, cat. no. 529552, or equivalent)
0.24 g MgSO_4_ (20 mM; Sigma‐Aldrich, cat. no. M7506, or equivalent)
Distilled H_2_O to 100 ml
Mix well
Autoclave
Add 2 ml filter‐sterilized 20% (w/w) glucose (Sigma‐Aldrich, cat. no. G7528, or equivalent)
Store ≤1 month at 4°C
  Ensure medium is free of contamination before use; cloudiness or the presence of floating clumps may indicate (microbial) contamination.

### Strep‐Tactin HRP solution

5 ml Pierce Fast Blocking Buffer (Thermo Fisher Scientific, cat. no. 37575)
1 µl Strep‐Tactin HRP (IBA LifeSciences, cat. no. 2‐1502‐001)
Prepare fresh immediately before use and store at 4°C for three more incubations

### Strep‐Tag II purification binding buffer

8 g sodium chloride (136.893 mM; Sigma‐Aldrich, cat. no. S3014, or equivalent)
0.2 g potassium chloride (2.683 mM; Sigma‐Aldrich, cat. no. 529552, or equivalent)
1.44 g sodium phosphate dibasic (10.144 mM; Millipore, cat. no. 567547, or equivalent)
0.245 g potassium phosphate monobasic (1.8 mM; Millipore, cat. no. 529568, or equivalent)
Distilled H_2_O to 0.8 L
Adjust to pH 7.4 by adding either HCl or NaOH
Mix well
Distilled H_2_O to 1 L
Filter using 0.45‐µm filter
Degas using vacuum filtration unit
Store ≤1 month at 4°C

### Strep‐Tag II purification elution buffer

8 g sodium chloride (136.893 mM; Sigma‐Aldrich, cat. no. S3014, or equivalent)
0.2 g potassium chloride (2.683 mM; Sigma‐Aldrich, cat. no. 529552, or equivalent)
1.44 g sodium phosphate dibasic (10.144 mM; Millipore, cat. no. 567547, or equivalent)
0.245 g potassium phosphate monobasic (1.8 mM; Millipore, cat. no. 529568, or equivalent)
0.53 g d‐desthiobiotin (2.5 mM; Sigma‐Aldrich, cat. no. D1411, or equivalent)
0.29 g EDTA (1 mM; Sigma‐Aldrich, cat. no. E9884, or equivalent)
Distilled H_2_O to 0.8 L
Adjust to pH 7.4 by adding either HCl or NaOH
Mix well
Distilled H_2_O to 1 L
Filter using 0.45‐µm filter
Degas using vacuum filtration unit
Store ≤1 month at 4°C

### TEAB, 100 mM

1 ml 1 M triethylammonium bicarbonate (TEAB) buffer (Sigma‐Aldrich, cat. no. 18597)
9 ml HPLC‐grade H_2_O
Store ≤1 week at 4°C

### TFA, 10%

1 ml 25% (v/v) trifluoroacetic acid (TFA; Sigma‐Aldrich, cat. no. 1.08218)
1.5 ml HPLC‐grade H_2_O
Store ≤1 month at room temperature

### Trypsin, 10 ng/µl

Add 20 µl 50 mM acetic acid to 20 µg lyophilized trypsin (Thermo Fisher Scientific, cat. no. 90057) to final 1 mg/ml trypsin
Add 100 ml 100 mM TEAB (see recipe) to dilute 100×
Prepare fresh immediately before use

## COMMENTARY

### Background Information

The development of innovative biosensors based on protein‐protein interactions (PPIs) has intensified the demand for precise characterization of binding affinity and kinetics. SPR is a robust, label‐free gold standard for these measurements, providing real‐time data on the association and dissociation rates of molecular pairs (Capelli et al., 2023). However, the complexity of experimental variables, ranging from sensor surface chemistry to mass transport limitations, often complicates the establishment of a streamlined, reproducible protocol for novice users.

Furthermore, although high‐throughput SPR platforms are increasingly common, many laboratories continue to rely on traditional instruments, such as the Biacore T100, which features a limited four‐flow‐cell architecture. This hardware constraint poses a challenge when the simultaneous analysis of numerous binder candidates is required (Feng et al., 2024; Yang et al., 2016). To address the need for efficient screening of large binder libraries, we present a validated SPR workflow for the comparative kinetic analysis of nanobody variants, using lysozyme as a model target. By following this step‐by‐step implementation, researchers can navigate the technical nuances of assay design and data fitting. When utilized in conjunction with the reference plasmids available via the Addgene repository, this protocol enables the transition from experimental design to data acquisition in <1 month.

### Critical Parameters

#### Protein expression and purification

- Use cells with confirmed good transformation efficiency. In this case, use strains that promote oxidizing environments, such as Shuffle T7.
- Use protease‐free conditions during lysis.
- Use appropriate IPTG, temperature, and incubation duration conditions.

#### Protein identification

- To prevent environmental and human protein contamination, all steps should be performed with gloves and under clean working conditions.
- Using fresh antibody or Strep‐Tactin and chemiluminescent reagents is key for a good western blot signal.
- Complete gel destaining before dehydration.

#### SPR workflow

- It is essential to use the exact same buffer in which the samples were equilibrated as the running buffer. Even preparing a new batch following the same recipe may introduce differences that can compromise the experiment and lead to poor results.
- EDC and NHS must be freshly thawed or prepared to support proper chip surface activation and ligand immobilization.
- Ligand and analyte must be of highest purity to avoid noise, unspecific binding, and other artifacts.
- Do not skip chip activation and startup cycles.
- Double subtraction is key to remove nonspecific signals, involving subtracting both a reference channel and a blank injection, correcting for baseline drift, bulk refractive index changes, nonspecific binding, pressure fluctuations, and solvent effects.

#### Ligand immobilization

- Surface activation and ligand attachment are normally performed at temperatures ≥25°C. Higher temperatures might in some cases increase the immobilization level. Temperature‐sensitive ligands may require lower temperatures and prolonged contact times for surface activation (GE Healthcare, 2001).
- The concentrations of ligand solutions for immobilization are typically in the range of 10 to 50 µg/ml. Ligands with a low isoelectric point or inefficient surface attachment might require higher concentrations or the use of a different chip surface (GE Healthcare, 2001).
- The buffer pH should be at least 0.5 to 1 unit below the isoelectric point of the ligand. The ionic strength should be low, usually no more than 10 to 20 mM monovalent cations. Primary amine groups or other strong nucleophilic groups must be avoided for amine coupling. Reducing agents should be avoided for thiol coupling. For most proteins, coupling in 10 mM acetate buffer, pH 4.5 to 6, works well. However, ligand solutions should be prepared shortly before use because many proteins are relatively unstable at low pH and low ionic strength.
- Other parameters that may require optimization include the analyte flow rate and the association time. An appropriate analyte flow rate ensures homogeneous analyte distribution across the sensor surface, minimizes mass transport limitations, and improves the accuracy and reproducibility of the derived kinetic constants. The association time should be sufficiently long to allow reliable detection of binding, even for slow interactions, and identification of non‐ideal interaction behaviors, while remaining limited to avoid unnecessary sample consumption.

### Troubleshooting

Table 3 lists potential problems in nanobody expression and purification. Table 4 outlines potential issues encountered in execution of the SPR workflow.

**Table 3:** Troubleshooting Guide for Protein Expression and Purification

Problem	Possible cause	Solution
No expression or low yield	Bacterial cryo‐stock is old or there was loss of plasmid	Repeat bacterial transformation
	Induction, medium, or other conditions are not optimal	Perform expression test if any expression conditions are changed
	Lysis conditions unfavorable	Ensure lysis buffer components are fresh and in good condition; perform 1‐2 more lysis passages using a French press
Insoluble protein	Misfolding or aggregation	Lower temperature after induction and increase culture growth time
Impure protein	Unspecific binding during IMAC purification	Increase imidazole concentration in the binding and lysis buffers up to 50 mM

**Table 4:** Troubleshooting Guide for SPR Workflow

Problem	Possible cause	Solution
Noisy signals	Air bubbles, precipitates	Degas or spin down buffers and proteins
Significant bulk contributions	Inadequate buffer exchange	Dialyze the sample in the running buffer
Warning that “Kinetic constants out of instrument specifications”	Analyzing ultra‐high or ultra‐slow binders	Try different buffer compositions. Try a different chip. Reduce ligand density, adjust analyte concentrations, change flow rate, or scout association and dissociation times. Alternatively, measure steady‐state affinity instead of kinetic rates.

### Understanding Results

#### Protein expression and purification

Nanobodies were produced in *E. coli* SHuffle T7 competent cells (Basic Protocol 1). Expression and purification were adapted from the protocol described by Birchenough et al. (2021) with modifications to the *E. coli* strain, culture medium, and induction process. SHuffle T7 is particularly suited for the cytoplasmic production of disulfide‐bonded proteins, as it provides an oxidizing intracellular environment and expresses the disulfide isomerase DsbC. Given that the H04 protein contains one disulfide bond (Cys23‐Cys97) and cAbLys3 protein contains two disulfide bonds (Cys34‐Cys110, Cys23‐Cys97), SHuffle T7 cells were used instead of the T7 Express strain previously employed (Birchenough et al., 2021). We opted to use LB medium and controlled IPTG induction in this protocol as a cost‐effective alternative to the Magic medium previously reported, while keeping induction incubation for 24 hr at 28°C. Under the conditions described in this protocol, nanobody yields varied between 0.1 and 8.4 mg per liter of culture (Table 5). Optimization of post‐induction incubation time and temperature may further improve yields, but this still would need to be tested.

**Table 5:** Nanobody Yields From 1 L Culture After IMAC and SEC Purifications

Construct name	Yield (mg/L cell culture)
H04‐6×His	8.4
H04‐Strep‐Tag II	1.0
cAbLys3‐6×His	2.8
cAbLys3‐Strep‐Tag II	0.1

**Figure 3:**
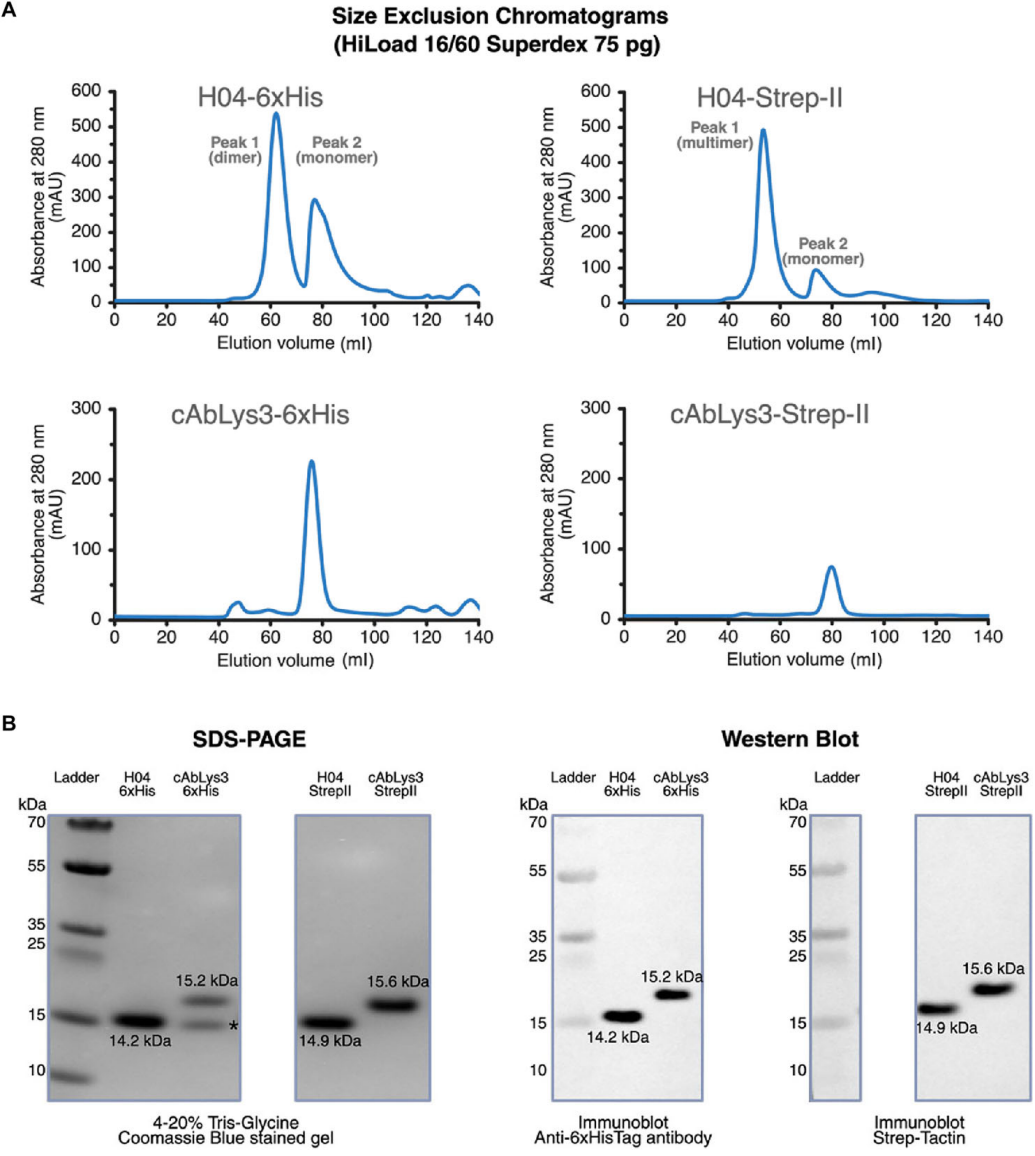
Protein expression, purification, and identification. (**A**) Size exclusion chromatography (SEC) traces of purified nanobodies. (**B**) Protein identification. Reducing SDS‐PAGE gel stained with Coomassie blue and western blot with 0.5 µg protein loaded are shown. For blotting, 6×His‐tagged samples were blotted with anti‐6×His antibody, and Strep‐Tag II‐tagged samples were blotted with Strep‐Tactin. The expected molecular weight is indicated over each band.

As previously reported by Birchenough et al. (2021), non‐reversible multimers were observed during purification of both versions of the H04 nanobody. The Strep‐Tag‐II‐tagged variant showed a markedly higher tendency to multimerize, with ∼83% of the total yield present in the multimeric form (Fig. 3A). Because it has been reported that the dimeric form of H04‐His does not bind lysozyme, only the monomeric fraction of H04‐His was collected for further experiments, whereas the monomeric and multimeric forms of H04‐Strep‐Tag II that were not previously tested were analyzed by SPR (see Supporting Information, Fig. S2).

Following SEC (Fig. 3A), all proteins analyzed by SDS‐PAGE were further characterized by western blot (Fig. 3B) and LC‐MS/MS (Support Protocol 1), confirming their amino acid sequences (see Supporting Information, Fig. S1).

#### SPR comparative analysis of lysozyme‐binding nanobodies

**Figure 4:**
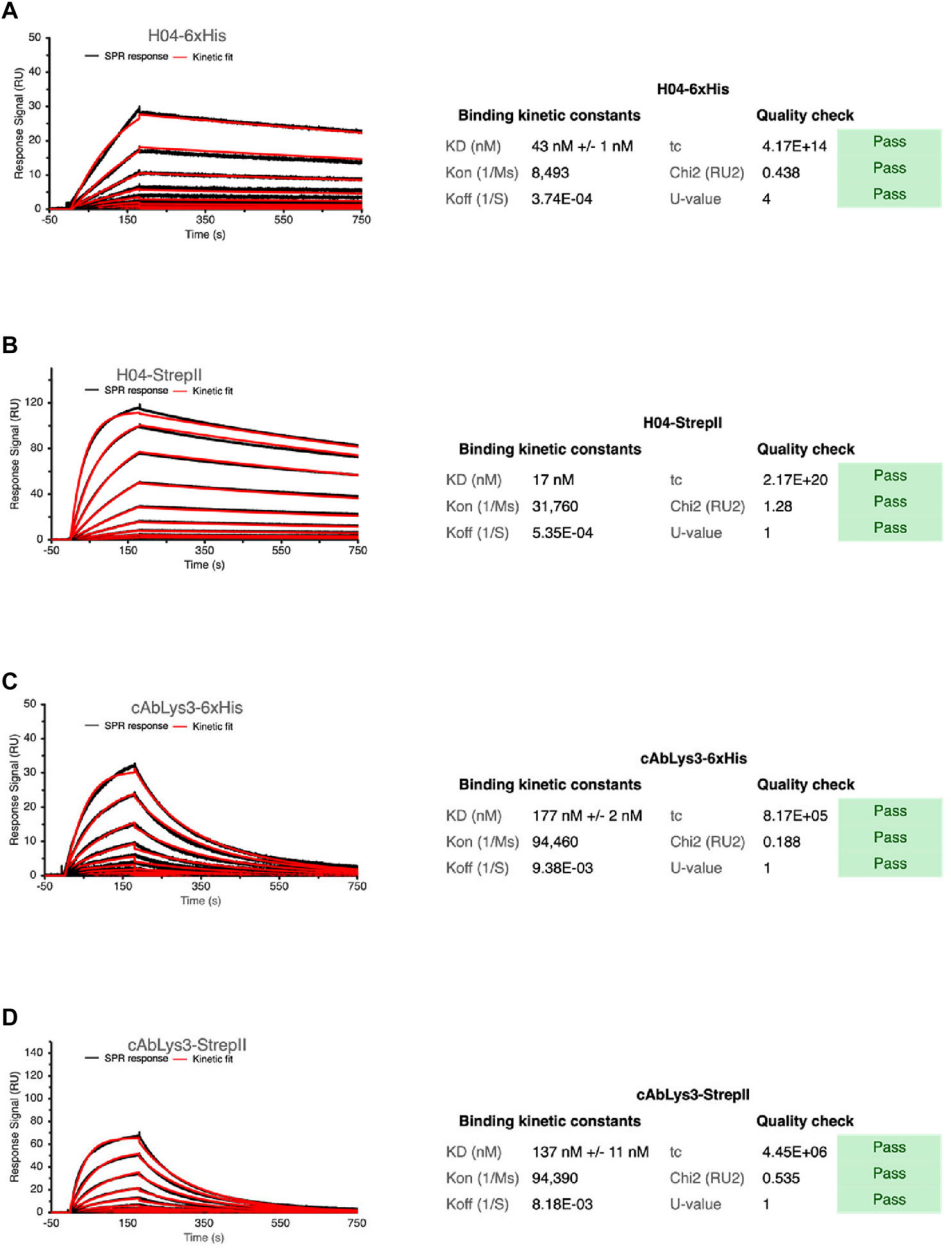
Surface plasmon resonance (SPR) sensograms with fit and quality check. (**A**) H04‐6×His. (**B**) H04‐Strep‐Tag II. (**C**) cAbLys3‐6xHis. (**D**) cAbLys3‐Strep‐Tag II.

The binding kinetics of four anti‐HEWL nanobodies toward HEWL were analyzed in a single SPR run (Basic Protocol 2). The resulting sensograms showed that even though both nanobodies bind strongly (in the nM range) to their target, H04 has a slower dissociation rate compared to cAbLys3 (Fig. 4).

The calculated surface activity ranged from 20% to 76%, reflecting the fraction of immobilized ligand that is functionally available, which is typical for amine coupling. Importantly, all analytes were measured on the same ligand surface, under identical conditions, ensuring that relative *K*_D_ values accurately reflect differences in affinity rather than surface inconsistencies. Using a single chip eliminates inter‐chip variability, providing a robust and internally consistent platform for affinity ranking. All traces passed the required quality check.

This protocol suggests a workflow for the application of SPR to the ranking of lysozyme‐binding nanobody variants by affinity in a single assay. This protocol aims to facilitate and guide novel SPR users by the step‐by‐step implementation of an SPR assay. In combination with the provided plasmids available in the Addgene repository, this protocol can be put into action in <1 month, depending on the speed of LC‐MS/MS analysis and SPR instrument availability. To succeed in the application of this workflow, careful consideration of the mentioned critical parameters is indispensable. If unexpected behavior is observed, consult the Troubleshooting section. Although the protocol is customized for the model interaction, it provides additional information for its adjustment to study other interactions of interest (Support Protocol 2). Further theoretical background can be found on the Cytiva website or in the Biacore handbook (Biacore, 2005; Cytiva, 2020; GE Healthcare, 2001). By integrating these recommendations, users can adapt the assay to a wide range of targets and experimental needs.

### Time Considerations

#### Basic Protocol 1 (6 to 8 days after plasmid arrival)

- Plasmid request (1 week to 1 month): If the sequence is ordered from Addgene, the synthesis can take from 1 week to 1 month (depending on the provider's ordering queue). Additionally, time might be required for shipment and customs clearance.
- Protein expression (3 to 4 days): Bacterial transformation combined with culture growth up to the harvest step will take a total of 70 to 75 hr. This step can be delayed in the case where expression optimization is needed or there are problems with the bacterial transformation/stock.
- Protein purification (3 to 4 days): From cell lysis to final protein concentration takes ∼2 days. Proteins can be processed in parallel, so producing all four variants requires 2 to 4 days. Concentration steps and SEC column equilibration, running, and cleaning time should be carefully considered for efficient usage of time.

#### Support Protocol 1 (3 days + LC‐MS/MS service time)

- Protein identification by SDS‐PAGE (3 to 4 hr): Commercially available gels support runs from 20 min to 1 hr and staining and destaining take ∼1 hr each.
- Protein identification by western blot (2 to 14 hr): This mostly depends on the desired length of the antibody incubation time.
- Protein sequence confirmation by LC‐MS/MS (2 days + LC‐MS/MS service): This mostly depends on the availability of the LC‐MS/MS instrument/service.

#### Basic Protocol 2 (>3 days)

- SPR sample preparation (2 to 6 hr to overnight): Buffer preparation and sample buffer exchange can be performed in the morning on the experimental day or a day ahead.
- Instrument setup (3 hr to overnight): Switching on the instrument and equilibration require time.
- Ligand immobilization (2 to 4 hr): This depends on the number of channels to immobilize and the optimized ligand concentration and flow as well as the ligand immobilization strategy. For two channels, reserving about 2 to 4 hr is enough.
- SPR assay setup (1 hr): Once the instrument, buffer, and samples are prepared, setting up the plate is fast.
- SPR run (19 hr): The SPR is usually run overnight. The duration of the run depends on the number of replicates and samples to be analyzed.

#### Support Protocol 2 (several days)

- Assay development (variable): Assay development could take several days depending on the number of iterations required. At least 1 to 2 days is required for initial scouting. Development possibly also requires 2 to 5 days for optimizing binding conditions before the assay.

### Author Contributions

**Escarlet Díaz‐Galicia**: Formal analysis; software; investigation; methodology; writing—original draft; data curation; validation; visualization. **Nicoleta Gutu**: Writing—original draft; investigation; formal analysis; data curation; validation; visualization. **Yuli Peng**: Writing—original draft; investigation; formal analysis; data curation; validation; visualization. **Almira Valitova**: Investigation; data curation; validation; writing—review and editing. **Dominik Renn**: Conceptualization; project administration; methodology; formal analysis; validation; investigation; writing—review and editing. **Magnus Rueping**: Conceptualization; funding acquisition; methodology; supervision; writing—review and editing.

### Conflict of Interest

The authors have declared no competing interests.

## Supporting information

Figure S1 Amino acid sequence confirmation based on tryptic digest results from LC‐MS/MS.Figure S2 H04‐Strep‐Tag II‐Peak1 analysis.Figure S3 Primary sequences of all proteins described in the article.

## Data Availability

All plasmids are available through the Addgene plasmid repository: pET29b_HEWL‐VHH‐cAbLys3‐6×His_1XFP (Addgene, no. 248353); pET29b_HEWL‐VHH‐H04‐6×His_4U3X (Addgene, no. 248574); pET29b_Strep‐HEWL‐VHH‐cAbLys3‐Strep‐Tag II_1XFP (Addgene, no. 248575); and pET29b_Strep‐HEWL‐VHH‐H04‐Strep‐Tag II_4U3X (Addgene, no. 248576). The SPR methods are available as follows: ImmobilizationWizard.bwImmob and KineticAssay_PBST_CM5_Lys_LysNbs.Method.
