# Supplementary material for: Surface Plasmon Resonance (SPR) Workflow for Comparative Analysis of Nanobody Variants Binding to Lysozyme as a Model Ligand
Source: Curr Protoc. 2026 Apr 29;6:e70360. doi: 10.1002/cpz1.70360 (PMC13127243; doi:10.1002/cpz1.70360)
Supplement: Supplementary file 1 — Figure S1 Amino acid sequence confirmation based on tryptic digest results from LC‐MS/MS.Figure S2 H04‐Strep‐Tag II‐Peak1 analysis.Figure S3 Primary sequences of all proteins described in the article. [file CPZ1-6-0-s001.docx]

**SUPPORTING INFORMATION**

**Surface Plasmon Resonance workflow for comparative analysis of nanobody variants binding to lysozyme as a model ligand**

Escarlet Díaz-Galicia^1^, Nicoleta Gutu^1^, Yuli Peng^1^, Almira Valitova^1^, Dominik Renn^1^, Magnus Rueping^1,2^

^1^KAUST Catalysis Center (KCC), Division of Physical Sciences & Engineering, King Abdullah University of Science and Technology, KAUST, 23955 Thuwal, Kingdom of Saudi Arabia

^2^Institute for Experimental Molecular Imaging, University Clinic, RWTH Aachen University, Forckenbeckstrasse 55, D52074 Aachen, Germany

***Correspondence:** dominik.renn@kaust.edu.sa, magnus.rueping@kaust.edu.sa

**Table of Contents**

| Amino acid sequence confirmation  H04-StrepII-Peak1 analysis  Protein Sequences  **S1. Amino acid sequence confirmation**  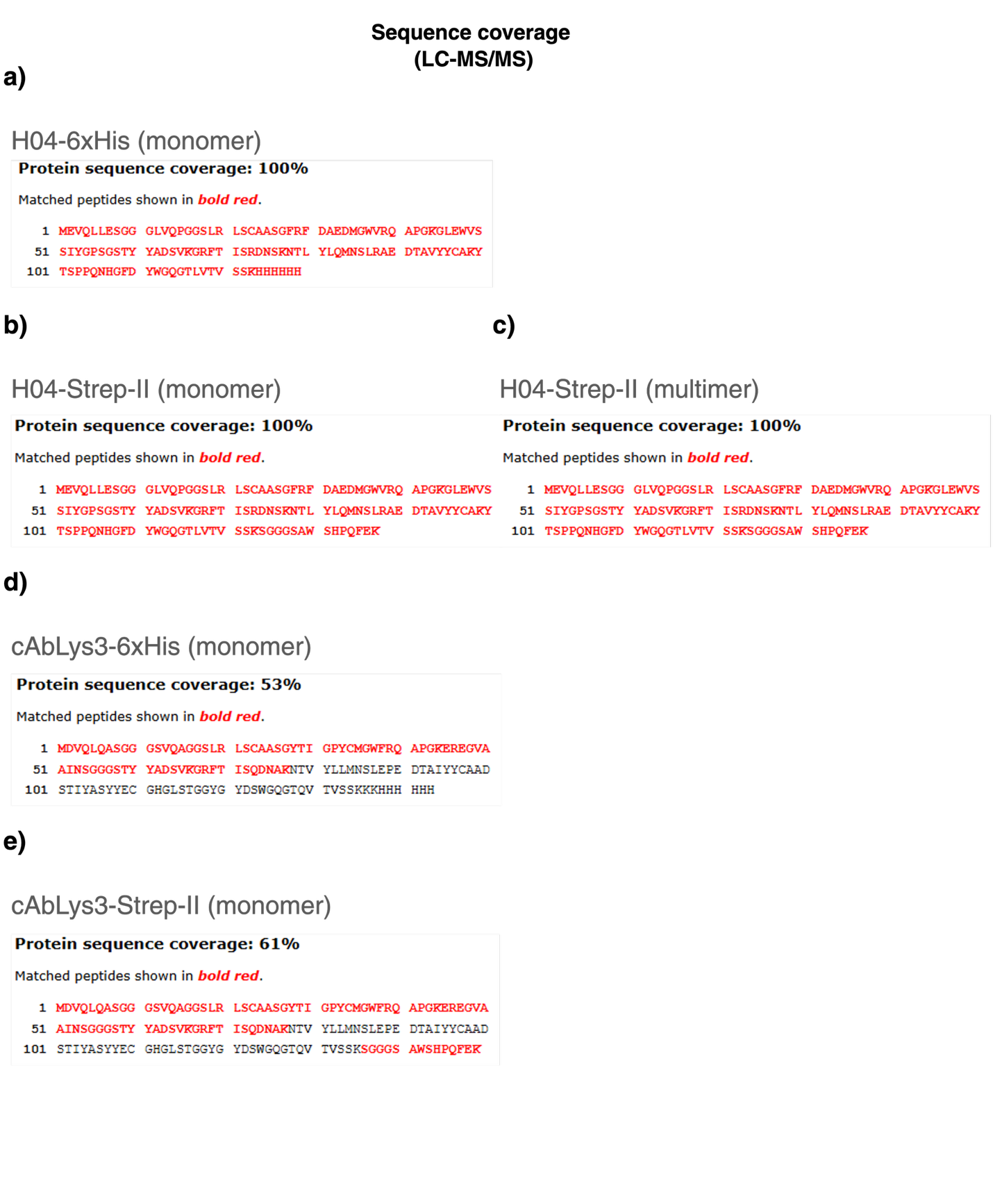  S1. Excerpt from the MASCOT summary report showing peptide coverage after tryptic digestion and LC-MS/MS identification of nanobodies.  **S2. H04-StrepII-P1 analysis**  Two populations of H04-StrepII were purified. Lower binding efficiency on the dimeric form was distinguishable though some binding remains unexpectedly (Figure S2).  **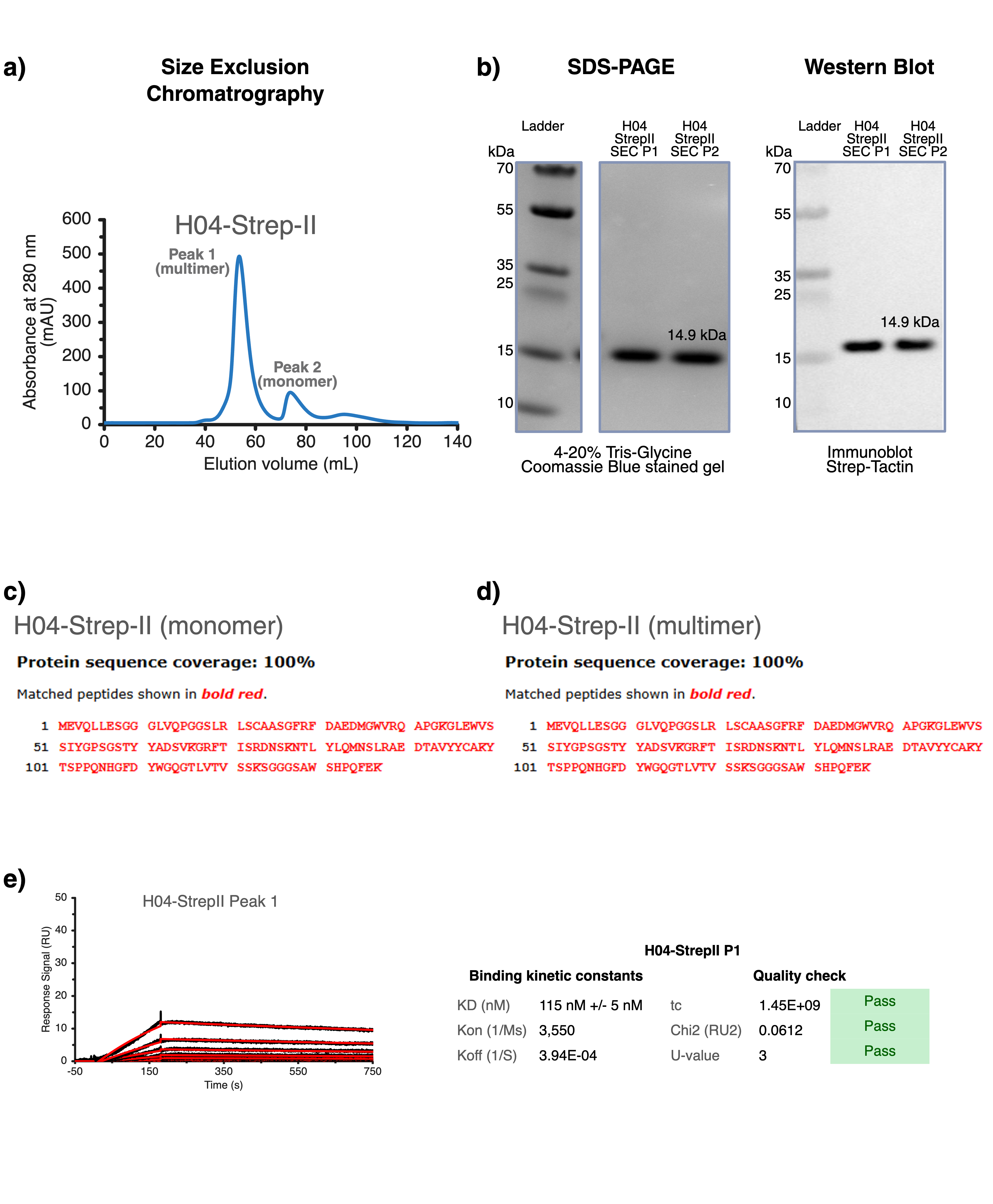** | S1  S2  S3 |
| --- | --- |

S2. H04-StrepII-Peak1 analysis. a) Size exclusion chromatogram showing two peaks of the H04-StrepII sample. b) Protein identification in SDS-PAGE both by Coomassie blue and Western blot. Protein sequence coverage by LC-MS/MS of c) H04-StrepII monomer and d) multimer. e) Binding sensogram, binding kinetic constants and quality check of H04-StrepII multimer (peak 1) by SPR.

**S3. Protein sequences**

>VHH-cAb-Lys3-6xHis_1XFP

MDVQLQASGGGSVQAGGSLRLSCAASGYTIGPYCMGWFRQAPGKEREGVAAINSGGGSTYYADSVKGRFTISQDNAKNTVYLLMNSLEPEDTAIYYCAADSTIYASYYECGHGLSTGGYGYDSWGQGTQVTVSSKKKHHHHHH

*Addgene cat. no. 248353

>VHH-H04-6xHis_4U3X

MEVQLLESGGGLVQPGGSLRLSCAASGFRFDAEDMGWVRQAPGKGLEWVSSIYGPSGSTYYADSVKGRFTISRDNSKNTLYLQMNSLRAEDTAVYYCAKYTSPPQNHGFDYWGQGTLVTVSSKHHHHHH

*Addgene cat. no. 248574

>VHH-cAb-Lys3-StrepII_1XFP

MDVQLQASGGGSVQAGGSLRLSCAASGYTIGPYCMGWFRQAPGKEREGVAAINSGGGSTYYADSVKGRFTISQDNAKNTVYLLMNSLEPEDTAIYYCAADSTIYASYYECGHGLSTGGYGYDSWGQGTQVTVSSKSGGGSAWSHPQFEK

*Addgene cat. no. 248575

>VHH-H04-StrepII_4U3X

MEVQLLESGGGLVQPGGSLRLSCAASGFRFDAEDMGWVRQAPGKGLEWVSSIYGPSGSTYYADSVKGRFTISRDNSKNTLYLQMNSLRAEDTAVYYCAKYTSPPQNHGFDYWGQGTLVTVSSKSGGGSAWSHPQFEK

*Addgene cat. no. 248576

**References**

Birchenough, H. L., Nivia, H. D. R., & Jowitt, T. A. (2021). Interaction standards for biophysics: Anti-lysozyme nanobodies. *European Biophysics Journal*, *50*(3), 333–343. https://doi.org/10.1007/s00249-021-01524-6
